# Increasing Capacity for the Treatment of Common Musculoskeletal Problems: A Non-Inferiority RCT and Economic Analysis of Corticosteroid Injection for Shoulder Pain Comparing a Physiotherapist and Orthopaedic Surgeon

**DOI:** 10.1371/journal.pone.0162679

**Published:** 2016-09-15

**Authors:** Darryn Marks, Leanne Bisset, Tracy Comans, Michael Thomas, Shu Kay Ng, Shaun O’Leary, Philip G. Conaghan, Paul A. Scuffham

**Affiliations:** 1 Gold Cost Hospital and Health Service, Gold Coast University Hospital, 1 Hospital Boulevard, Southport, 4215, Gold Coast, Australia; 2 Menzies Health Institute Queensland, Griffith University, Parklands Drive, Southport, QLD 4222, Gold Coast, Australia; 3 Metro North Hospital and Health Service, 112 Alfred Street, Fortitude Valley, QLD 4006, Brisbane, Australia; 4 School of Health and Rehabilitation Sciences, University of Queensland, Brisbane, St Lucia, QLD 4072, Australia; 5 Physiotherapy Department, Royal Brisbane and Women’s Hospital, Butterfield Street, Herston, QLD 4006, Australia; 6 Leeds Institute of Rheumatic & Musculoskeletal Medicine, University of Leeds, & NIHR Leeds Musculoskeletal Biomedical Research Unit, Chapel Allerton Hospital, Leeds, LS7 4SA, United Kingdom; University of South Australia, AUSTRALIA

## Abstract

**Background:**

Role substitution is a strategy employed to assist health services manage the growing demand for musculoskeletal care. Corticosteroid injection is a common treatment in this population but the efficacy of its prescription and delivery by physiotherapists has not been established against orthopaedic standards. This paper investigates whether corticosteroid injection given by a physiotherapist for shoulder pain is as clinically and cost effective as that from an orthopaedic surgeon.

**Methods:**

A double blind non-inferiority randomized controlled trial was conducted in an Australian public hospital orthopaedic outpatient service, from January 2013 to June 2014. Adults with a General Practitioner referral to Orthopaedics for shoulder pain received subacromial corticosteroid and local anaesthetic injection prescribed and delivered independently by a physiotherapist or a consultant orthopaedic surgeon. The main outcome measure was total Shoulder Pain and Disability Index (SPADI) score at baseline, six and 12 weeks, applying a non-inferiority margin of 15 points. Secondary outcomes tested for superiority included pain, shoulder movement, perceived improvement, adverse events, satisfaction, quality of life and costs.

**Results:**

278 participants were independently assessed by the physiotherapist and the orthopaedic surgeon, with 64 randomised (physiotherapist 33, orthopaedic surgeon 31). There were no significant differences in baseline characteristics between groups. Non-inferiority of injection by the physiotherapist was declared from total SPADI scores at 6 and 12 weeks (upper limit of the 95% one-sided confidence interval 13.34 and 7.17 at 6 and 12 weeks, respectively). There were no statistically significant differences between groups on any outcome measures at 6 or 12 weeks. From the perspective of the health funder, the physiotherapist was less expensive.

**Conclusions:**

Corticosteroid injection for shoulder pain, provided by a suitably qualified physiotherapist is at least as clinically effective, and less expensive, compared with similar care delivered by an orthopaedic surgeon. Policy makers and service providers should consider implementing this model of care.

**Trial Registration:**

Australia and New Zealand Clinical Trials Registry 12612000532808

## Introduction

Musculoskeletal disorders are the second largest cause of disability globally [1, 2]. In countries with publicly funded health systems, long waiting lists for specialist musculoskeletal care such as orthopaedics, attract much political attention [3] and service redesign effort [4]. In response, funding and service modernization in England has led to some improvement but up to 5% of patients still wait beyond recommended periods [5]. In many countries the response has been slower, for example patients seeking orthopaedic consultation in Australia’s public hospitals often wait in excess of 12 months [6]. Internationally, access problems are expected to intensify as the rate of musculoskeletal disorders rise with population ageing [7], causing increased demand for care and mounting challenges for service providers [4, 8, 9].

A strategy aiming to improve access for patients [10], and to reduce cost and workforce shortages [11], is the substitution of doctors with other healthcare professionals. It has been proposed that lower costs, reduced waiting times and improved health outcomes may be achieved when extended-scope physiotherapists provide various aspects of musculoskeletal care in place of doctors [12–14]; however, the supporting evidence is generally low quality with conclusions drawn mostly from observational case reports rather than robust scientific investigation [12, 14]. With a lack of high quality evidence to inform service redesign, regulations, funding and delivery structures have evolved with substantial international variability, highlighted by the legalisation of independent prescribing by trained physiotherapists in the UK [15, 16], but not in other countries.

Shoulder pain is a common musculoskeletal disorder that frequently highlights a discrepancy between the evidence (which generally advocates non-surgical treatment) and health service delivery (which frequently directs patients to orthopaedic surgery waiting lists). It is the second to third most prevalent musculoskeletal condition [17–20], causes substantial physical, social and psychological deficits [21, 22], reduced ability to work and high levels of work absence [23, 24]. The quality of primary care management of shoulder pain is variable, with a high reliance upon specialist referral [25], most commonly to orthopaedics [26, 27]. Yet there is evidence that involving a physiotherapist in the triage of orthopaedic referrals may be beneficial [13, 28], and shoulder pain is a frequent problem seen by physiotherapists providing early access orthopaedic services [29]. Subacromial impingement syndrome (referred to by various terms including rotator cuff disease) is the most common cause [30, 31], and should generally be managed non-surgically, unless symptoms persist despite best conservative efforts [32–34]. There are a variety of treatment options for subacromial impingement [32], and whilst not always indicated as the initial treatment, both subacromial corticosteroid injection [32, 35, 36] and exercises [32, 37] are frequently recommended and cost effective [38, 39]. Therefore, it is possible that care may be expedited and possibly enhanced, with a medical substitution model permitting patients to access trained physiotherapists capable of providing these injections. This model of care is now available in the UK but the efficacy remains unknown as no clinical trials have yet investigated health outcomes resulting from prescribing or shoulder injection provided by physiotherapists compared with consultant level doctors or general practitioners. Furthermore, the lack of evidence surrounding the safety, efficacy and cost of prescribing and injection by physiotherapists may also be preventing other countries from adopting this innovative care model with the potential to improve patient access to evidence based care.

Therefore, the purpose of this this study was to determine if corticosteroid injection for shoulder pain, prescribed and delivered by a physiotherapist, is at least as clinically effective as that by a consultant orthopaedic surgeon. To test this hypothesis we chose a non-inferiority design, as it is the most appropriate way to investigate a new treatment in comparison to an existing gold standard [40, 41]. Secondary aims were to compare the safety, satisfaction and cost of this care delivered by a physiotherapist compared with an orthopaedic surgeon.

## Methods

### Design

A double blind (patient and outcome assessor) non-inferiority randomized controlled trial was undertaken in the Gold Coast, Australia, as described in the published protocol [42]. Other elements within this protocol (investigation of the economic burden of shoulder pain and the level of decision-making agreement between a physiotherapist and orthopaedic surgeon) will be reported separately.

### Approvals and governance

The trial was registered on the Australia and New Zealand Clinical Trials Registry 21 May 2012: 12612000532808. Ethical Approval was granted through the Gold Coast Hospital and Health Service Human Research Ethics Committee, NHMRC code EC00160 (HREC/12/QGC/30; SSA/12/QGC/97), and Griffith University Human Research Ethics Committee (MED/23/13/HREC). Legislative Approval was obtained under Queensland’s Health (Drugs and Poison) Regulation 1996, to permit prescribing / injection by the research physiotherapist, as this practice is beyond the usual professional scope of physiotherapy in Australia. Adverse event reporting and management, and the function of the Data Safety Monitoring Committee, are detailed in the published protocol [42].

### Patients

Adults aged 18 years or older were recruited between January 2013 and June 2014, providing they had a shoulder pain referral from their primary care doctor to the hospital orthopaedic department, the ability to read trial literature and give consent. They were excluded if they had prior knowledge of the research physiotherapist or the orthopaedic surgeon (eg, from previous interactions or consultation), or no shoulder X-Ray in the previous 12 months (in keeping with hospital referral guidelines). Following an initial telephone call, potentially eligible patients were offered a clinic appointment at which eligibility was confirmed, and written informed consent obtained by the research assistant. Prior to randomization, participants were independently assessed by the orthopaedic surgeon and by the physiotherapist, on a 30-minute clinic schedule per clinician, in variable order (orthopaedic surgeon or physiotherapist first) according to clinician availability. Participants were ineligible for randomization if either clinician’s assessment revealed that they were taking anticoagulant medication, required prophylactic antibiotics prior to injection, were pregnant, breastfeeding, or had previous surgery of the involved shoulder. Each clinician recorded their diagnosis, and a yes/no response to the question “would you provide subacromial injection today?” These were compared by the research assistant, and only if both clinicians recorded a “yes” response, was the participant eligible for randomization. Thus all randomized participants had (from both assessors) a diagnosis of subacromial impingement, and were deemed appropriate for immediate corticosteroid injection. Those not meeting this criteria, took no further part in the study. Providing the participant could complete the study questionnaires and follow post injection instructions, written informed consent to treatment in the study was obtained before they were randomized. In bilateral presentations, the painful shoulder identified in the referral was treated. In bilateral referrals both sides were assessed, the participant asked to nominate their most troublesome side and clinicians made their injection decisions for the most troublesome shoulder only. Patients were free to withdraw from the trial at any point, and receive usual orthopaedic care.

### Blinding

The research physiotherapist (DM) and the orthopaedic surgeon (MT) each had their own room labelled “room A” or “room B”, in which all assessments and injections were conducted independently. Both were blind to each other’s clinical assessment findings and treatment decisions. Participants were blind to the profession of surgeon and the physiotherapist at all times throughout the entire trial. The outcome assessor was blind to the treating practitioner. Post injection, treatment physiotherapists were asked to not seek information regarding the profession of the injecting clinician, not discuss the identity or profession of injecting clinicians with participants and to report any breaches in blinding to the research assistant. The success of blinding was assessed immediately post injection, and at 6 and 12 weeks, when participants and the outcome assessor nominated whether they thought the injection was delivered by the physiotherapist or the surgeon. The research physiotherapist (DM) and orthopaedic surgeon (MT) took no part in the recruitment, consent, randomization, or data collection.

### Randomisation

Using excel software, a computer generated randomization sequence was developed by one investigator (LB) and concealed from the research physiotherapist, orthopaedic surgeon and outcome assessor. Randomized participants were given a previously sealed opaque envelope by the research assistant, which contained a form directing them to either “room A” or “room B” for their injection.

### Interventions

#### Injection

Subacromial corticosteroid and local anaesthetic (one ml of Betamethasone (Celestone Chronodose 5.7mg/mL) mixed with five ml of 1% lignocaine hydrochloride), was delivered to the subacromial space using an aseptic injection technique by either the physiotherapist (intervention) or the orthopaedic surgeon (control) according to the randomization schedule. Subacromial corticosteroid injection is a common treatment for shoulder pain [43, 44], with evidence of clinical efficacy [35, 36] and cost effectiveness [38, 45]. Post injection, standard advice was provided verbally and participants were also given written information advising reduced activity for one week, attendance at physiotherapy and information pertaining to the recognition and reporting procedures for any adverse reactions.

#### Physiotherapy

All participants were referred for a course of physiotherapy within the hospital’s outpatient physiotherapy department, beginning approximately one week post-injection. The research (injecting) physiotherapist (DM) took no part in this course of post-injection treatment. Consensus regarding best practice exercise and manual therapy interventions used for shoulder pain in the department was established pre-trial and treatment was delivered pragmatically, with specific interventions and the number of sessions at the discretion of the treating physiotherapist. All treating physiotherapists were directed to not deliver acupuncture to participants and adherence to this was monitored by the research assistant.

#### Clinicians providing injections

The research physiotherapist (DM) had 19 years post-graduation experience in a variety of musculoskeletal settings, a post-graduate Diploma in injection therapy and Non-Medical Prescribing registration obtained in the UK. The orthopaedic surgeon (MT) was 20 years post-graduation, employed as a Staff Specialist Orthopaedic Consultant and was a Fellow of the UK Royal College of Surgeons (Trauma and Orthopaedics), and in 2005 was the recipient of an Australian Upper Limb Orthopaedic Fellowship.

#### Outcome measures

In addition to participant demographics, the usual waiting time for an initial appointment with an orthopaedic surgeon and for an appointment with an orthopaedic screening service physiotherapist, were taken at baseline from hospital databases. The primary outcome measure was the total Shoulder Pain and Disability Index (SPADI) score measured at baseline, 6 and 12 weeks post injection. This self-rating tool has demonstrated good responsiveness [46], reliability and validity in shoulder pain trials [47, 48], including the efficacy of corticosteroid injection [49, 50]. It uses a one week recall period and has 13 questions with a scale of 0–10, divided into pain (five questions) and disability (eight questions). These are scored as two separate subscales and an overall score varying from 0 (best) to 100 (worst) is calculated [48]. A number of secondary outcomes were also measured at initial attendance, six and twelve weeks. Pain severity over the past three days was measured on a visual analogue scale (VAS). The pain VAS has demonstrated reliability, validity and responsiveness to change in relevant populations [51, 52]. Health related quality of life was measured using the European Quality of Life five dimensions, five levels (EQ-5D-5L). This is recommended as a generic patient reported outcome measure for use with musculoskeletal populations [53] and has demonstrated validity and responsiveness in chronic pain [54]. A five-point Global Rating of Change (GROC) scale varying from completely recovered (1) to much worse (5) was also used. GROC is a convenient clinical measurement tool with demonstrated validity and responsiveness [55]. Within a period of 20 minutes before and 20 minutes after injection, the blinded outcome assessor also measured shoulder range of motion (ROM) three times in the scapular plane with a goniometer, by instructing the participant to “take your arm up as far as you feel able”. Goniometry for shoulder ROM has good validity and reliability [56] and intra-rater change detection of six degrees [57]. Participants also rated the severity of their pain (‘worst pain’ and ‘mean pain’) during shoulder elevation on a 0–100 VAS anchored by “no pain” (0) and “worst pain imaginable” (100). Medicine use was recorded at baseline and twelve weeks. Participants were prompted to report any unwanted side-effects via written instructions on a standardised post injection information sheet and were also asked by the research assistant at six weeks whether they had suffered any adverse events. A serious adverse event was defined as one that results in persistent incapacity, requires hospitalization (for example antibiotic treatment for infection related to the injection), or anything worse [58, 59]. Any adverse events reported over the twelve weeks post injection were recorded. At twelve weeks participants also rated their overall satisfaction with care on a 0–10 VAS anchored by “not satisfied at all” (0) to “completely satisfied” (10). The number of post injection physiotherapy sessions was obtained from hospital booking systems.

### Sample size calculation

**Fig 1:**
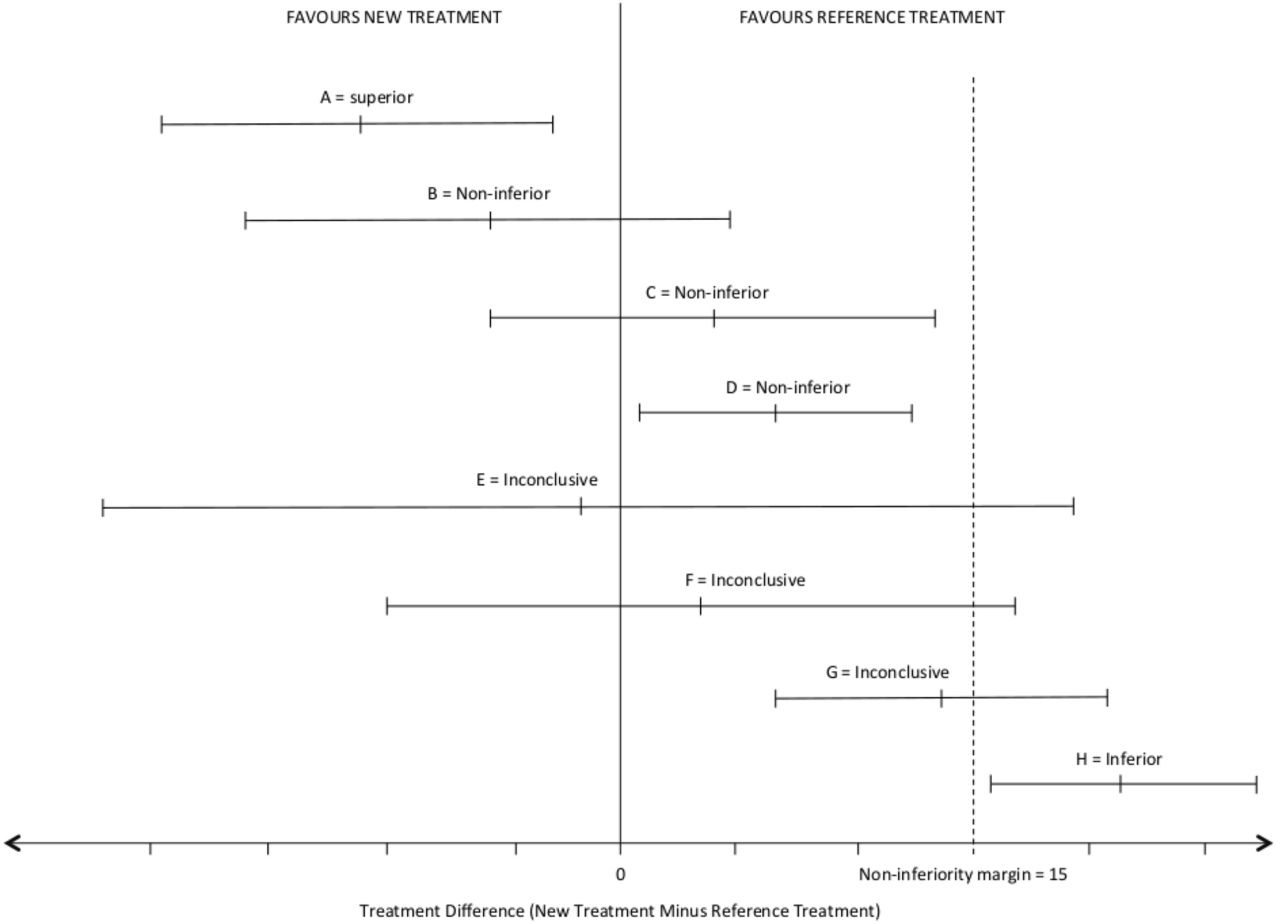
Possible scenarios of treatment differences and interpretation of non-inferiority results. Error bars indicate two sided (1–2 α) x 100% confidence intervals, where α is the Type I error rate. The zone of non-inferiority is to the left of the non-inferiority margin. Scenario A, new treatment is superior as the CI lie to the left of zero. Scenarios B and C, new treatment is non-inferior but not superior as the CI lie to the left of the non-inferiority margin and include zero. Scenario D, represents an unusual circumstance in which the new treatment is both non-inferior (as defined by the non-inferioirty margin), and inferior (as defined by exclusion of the null treatment difference). This can result from a very large sample size, or a non-inferiority margin that is too wide. Scenarios E and F, the difference is non-significant but non-inferiority is inconclusive. Scenario G, represents a significant result but non-inferioirty is inconclusive. Scenario H, inferior as the CI lie to the right of the non-inferiority margin. Adapted from Piaggio et al 2012 and 2006 [41, 60].

Assuming α = 0.05, β = 0.2, a non-inferiority margin of 15 points in total SPADI scores, we estimated a sample size of 64 participants (32 per group) to allow for dropouts, would be required to test for comparative efficacy between the intervention (injection by physiotherapist) and the control (injection by orthopaedic surgeon) groups. Detailed explanation of the sample size calculation, and non-inferioirty margin selection, are contained in the published protocol [42]. By using the generalised estimating equation (GEE) approach for repeated measures, this sample size provides the study with an increased power of above 90%. Fig 1 demonstrates the conceptual basis of non-inferiority design, and with reference to Fig 1, it means that the difference in mean SPADI scores between the two groups should be at most 5 points in order that the upper limit of the one-sided 95% confidence interval is smaller than the selected non-inferiority margin (Scenario C). We anticipated 256 participants would be assessed to achieve this, based on an estimate of 25% of participants being randomized.

### Statistical Analysis

Data analysis was conducted using SPSS software version 22, with the analyst (DM) blinded to the identity of the treatment and control groups (labelled as group 0 and group 1). All tests were conducted with an alpha of 0.05, on an intention to treat (ITT) basis and non-inferiority tests were performed in both directions (group 0 relative to group 1, and vice-versa). The analyst was then unblinded to determine the per-protocol population and analysis. Per-protocol analysis was compared to ITT analysis for non-inferiority tests due to the increased risk of type 1 error with ITT analysis in non-inferiority design [41, 60, 61].

For the primary outcome (SPADI) the intervention (physiotherapist) was tested for non-inferiority compared to the control (orthopaedic surgeon) using the generalized estimating equation (GEE) with a first order autoregressive working structure (AR1) and robust estimator for covariance matrix. Time (within-group differences), practitioner (between-group differences), and practitioner by time interaction (between-group differences over time) were included in all models and assessed using the Wald chi-square test. The GEE is a widely used method for the analysis of longitudinal data [62]. It considers multiple time points simultaneously and allows for testing the overall significance of the effects. The GEE works well with missing data, assuming that they are missing completely at random (MCAR). Non-linear relationship between outcome and time was considered in the analysis. We included baseline demographic characteristics (age, duration of symptoms, number of prior injections) as covariates in the omnibus analysis and reported unadjusted results if they were found to not affect outcomes significantly. Non-inferiority of injection by the physiotherapist could be declared if the upper limit of the 95% one-sided confidence interval for the difference in mean change of SPADI in the physiotherapist group, relative to the surgeon group, was smaller than the non-inferiority margin of 15 points [40, 63]. Secondary outcome measures were compared for superiority; between group change scores for pain and EQ-5D-5L health utility scores with independent t-tests. GROC was compared on the day of injection, 6 and 12 weeks using Mann-Whitney U. Changes in shoulder medication use at 12 weeks were categorised (1 = stopped, 2 = reduced, 3 = no change, 4 = increased) and analysed using Mann-Whitney U. Adverse events were presented descriptively, satisfaction with the treatment and the number of physiotherapy sessions were compared at 12 weeks using independent t-tests.

A within trial economic analysis with a 12 week time horizon, from the perspective of the health funder (Commonwealth and State governments), was undertaken and the incremental cost utility ratio estimated as: Cost-utility = (Cost_i—Cost_c) /(QALY_i—QALY_c), where: QALY = Quality adjusted life years calculated by mapping the EQ-5D-5L utility score across time and calculating the area under the curve, i = Intervention group for main effect analysis, and c = Control group. EQ-5D-5L scores were converted to Australian values using previously published Australian population data [64]. Inputs included direct staff costs derived from Queensland Government pay scales and total employment package costs to the employer [65, 66] for the injecting physiotherapist, orthopaedic surgeon and treating physiotherapists. A sample of 10,000 bootstraps was used to estimate the confidence interval around the incremental cost and effectiveness ratio. One-way sensitivity analyses were conducted to identify the key factors affecting the results, including physiotherapist pay rates and consultation time, support staff and a federal government reimbursement to the hospital, applicable only to consultation with a medical doctor [67].

## Results

**Fig 2:**
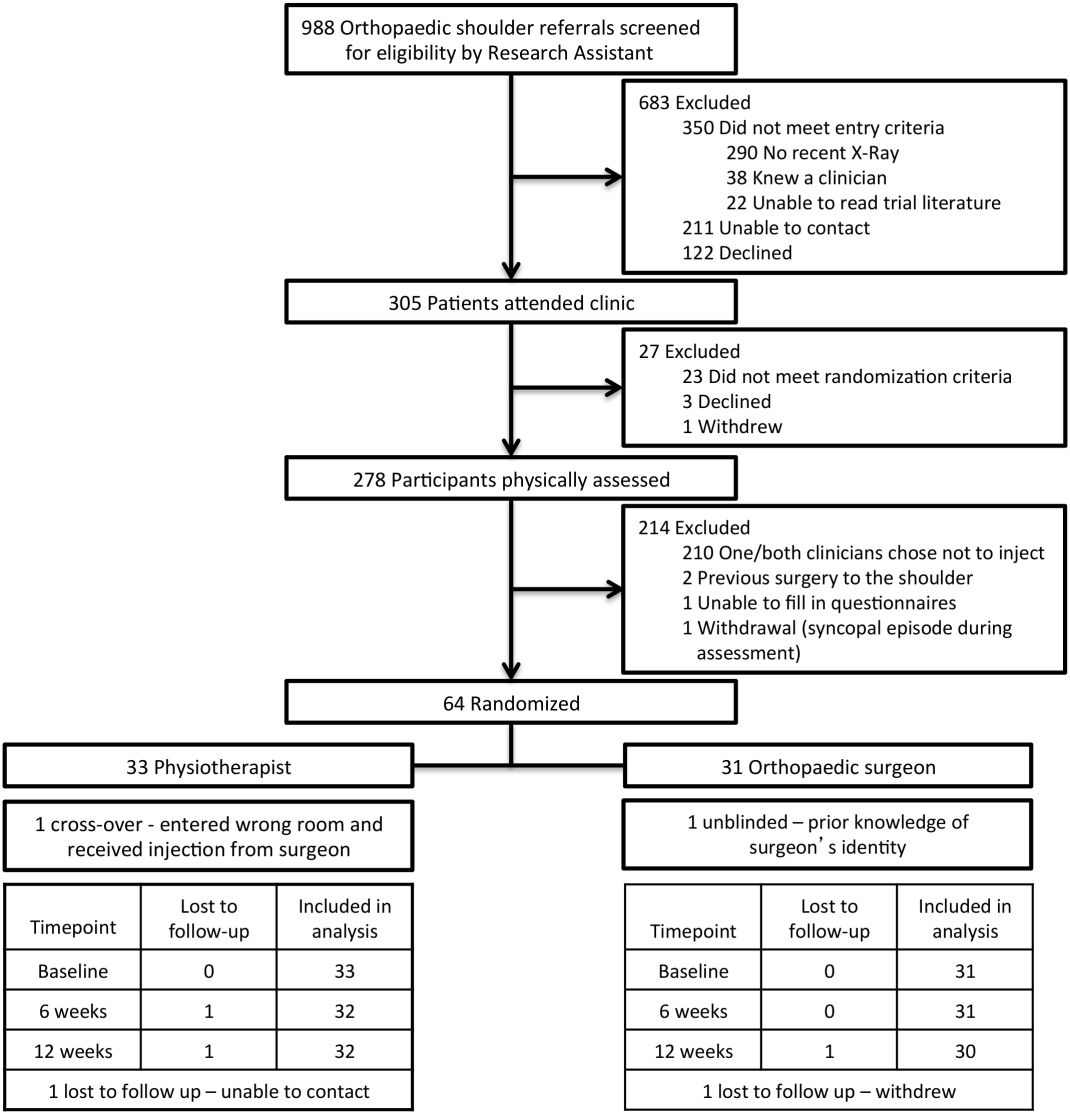
CONSORT flow diagram.

A total of 988 referrals on the orthopaedic waiting list were screened for eligibility by the research assistant. Of these, 305 attended a clinic appointment over the 17 month period between January 2013 and June 2014, of which 278 were assessed by the physiotherapist and surgeon before the target randomization sample size of 64 was achieved. This process and the reasons for exclusion are detailed in Fig 2.

Baseline characteristics were similar in both groups (Table 1) and did not significantly influence outcomes, thus unadjusted results have been reported. Although the orthopaedic surgeon group reported longer duration of symptoms, slightly higher SPADI scores and slightly more pain over the previous three days, there were no significant differences between the two groups in any baseline characteristics. The mean (and standard deviation) total SPADI score at baseline in the 64 randomized participants of 62.63 (19.07), was not significantly different to the 214 assessed but non-randomized patients at 56.97 (23.72), indicating that the inclusion criteria maintained external validity with respect to the severity of participant pain and disability. Compliance with the study protocol was high (Fig 2). One participant in the physiotherapist group reported difficult social circumstances, did not attend post injection physiotherapy or complete six or 12 week outcomes. One participant in the orthopaedic surgeon group withdraw from the trial and did not supply 12 week outcomes. Imputation of missing data was deemed unnecessary due to the low numbers with missing data and the use of the GEE, which accounts for missing observations [68]. Two participants (one from each group) were not included in the per-protocol analysis; one randomized to the physiotherapist group mistook the instruction within the sealed envelope, entered the wrong clinical room and received injection from the surgeon, instead of the physiotherapist. One other subject revealed they were un-blinded because they knew the surgeon from prior consultation with a family member. Both participants’ results were included within their allocated group according to the randomization schedule for the intention to treat analysis. Post-injection physiotherapy was intended to be pragmatically delivered and consequently low attendance was not considered a protocol breach.

**Table 1:** Baseline characteristics of randomised participants.

Baseline characteristics	Physio (n = 33)	Surgeon (n = 31)	p-value*
Age (years) mean (SD)	59.42 (10.49)	63.06 (10.07)	0.16
Gender female (%)	19/33 (57)	15/31 (48)	0.47
Duration of symptoms (months) median IQR	7 (4–18)	10 (4–24)	0.29^
Working (%)	17/33 (52)	15/31 (48)	0.81
Prior injections involved shoulder, mean (SD)	0.64 (0.70)	0.77 (0.85)	0.48
SPADI, mean (SD)	59.23 (19.90)	66.25 (17.74)	0.14
Worst pain last 3 days, VAS mean (SD)	60.00 (26.24)	69.26 (20.51)	0.12
Average pain last 3 days, VAS mean (SD)#	47.10 (26.05)	57.84(21.35)	0.09
EQ-5D-5L health utility score, mean (SD)	0.54 (0.27)	0.45 (0.28)	0.23

*Independent sample t-test with 95% confidence intervals

^ Independent samples Mann-Whitney U

^#^ data missing for 5 subjects

IQR = interquartile range, VAS = visual analogue scale

Participants in the physiotherapist group correctly guessed the profession of their injection provider 60% of the time at baseline, 68% at six weeks and 56% at 12 weeks. In the orthopaedic surgeon group, the profession of the injection provider was correctly guessed 68%, 71% and 60% at those time-points. This indicates participants were able to guess the clinician’s profession slightly more than would be expected by chance. It also indicates there was no systematic unblinding that occurred over the 12 week trial. The blinded outcome assessor (responsible for measuring range of movement within 20 minutes pre and post injection) correctly guessed the profession of the clinician providing injection in 79% and 74% of cases for the physiotherapist and surgeon group respectively.

### Primary outcome

Table 2 details the change in mean SPADI scores at 6 and 12 weeks compared to baseline. Mean total SPADI scores improved significantly in both groups at 6 and 12 weeks (p<0.001). Improvement was greater in the surgeon group at 6 weeks and greater in the physiotherapist group at 12 weeks but between group differences did not reach significance. Non-inferiority of the physiotherapist compared to the orthopaedic surgeon was declared at both 6 (Fig 1 scenario C) and at 12 weeks (Fig 1 scenario B), as the upper limit of the one-sided confidence interval fell below the non-inferiority margin of 15 SPADI points (13.34 and 7.17 at 6 and 12 weeks respectively). This outcome was consistent across intention to treat and per-protocol analyses. SPADI pain and disability subscale and change scores, at both 6 and 12 weeks revealed no significant differences between groups (data not shown, available from corresponding author).

**Table 2:** Primary outcome: total Shoulder Pain and Disability Index.

	Physio	Surgeon	t-test		GEE
	Change from baseline. Mean (SD)	Change from baseline. Mean (SD)	Difference in change score. Mean (two-sided 90% CI)*	p-value	Difference Physio—surgeon (two-sided 90%CI)*
**Intention to treat analysis**					
6 weeks	N = 32	N = 31			
	-22.46 (23.43)	-25.87 (22.48)	3.41 (-6.25 to 13.08)	0.59	3.96 (-5.41 to 13.34)*
12 weeks	N = 32	N = 30			
	-29.40 (31.04)	-24.46 (26.47)	-4.93 (-17.21 to 7.34)	0.50	-4.65 (-16.46 to 7.17)*
**Per-protocol analysis**					
6 weeks	N = 31	N = 30			
	-22.93 (23.66)	-25.84 (22.87)	2.90 (-7.06 to 12.86)	0.63	3.49 (-6.17 to 13.14)*
12 weeks	N = 31	N = 29			
	-31.14 (29.92)	-24.67 (26.91)	-6.47 (-18.87 to5.84)	0.38	-6.14 (-18.02 to 5.73)*

* Non-inferiority of the physiotherapist compared to the surgeon declared as the upper limit of the two-sided confidence interval (which is equivalent to the one-sided 95% confidence interval) does not exceed 15. This equates to scenario C (Fig 1) at 6 weeks, and scenario B (Fig 1) at 12 weeks. GEE = generalised estimating equation.

### Secondary outcomes

Table 3 details the secondary outcome measures. Hospital data showed that in usual service delivery, patients would have waited significantly longer to see the orthopaedic surgeon (356 days), than to see the physiotherapist (119 days). With the exception of the GROC on the day of injection (significant result in favour of the physiotherapist), there were no statistically significant differences between intervention groups at any point in terms of safety, clinical efficacy, patient satisfaction or resource usage (post-injection physiotherapy treatment and medication use). No serious adverse events were reported throughout the trial. One subject injected by the surgeon reported self-limiting symptoms of headache and mild confusion for approximately two weeks following injection. One subject injected by the physiotherapist reported symptoms of headache and a rash (torso). These symptoms resolved without treatment and as they had not appeared until two days after the injection, a causative relationship to the injection was not established.

**Table 3:** Secondary outcome measures.

		Physio	Surgeon		
	Week	Mean (SD)	Mean (SD)	Mean difference (95% CI)	p-value^+^
**Worst pain—change from baseline (100mm VAS)**	6	-10.58 (34.33)	-21.23 (26.71)	10.65 (-4.88 to 26.19)	0.18
	12	-25.97 (35.46)	-20.16 (37.73)	-5.8 (-24.41 to 12.78)	0.53
**Av. pain—change from baseline (100mm VAS)**	6	-14.87 (31.36)	-16.51 (25.61)	1.65 (-13.47 to 16.77)	0.82
	12	-22.55 (30.28)	-17.19 (30.90)	-5.36 (-21.62 to 10.89)	0.51
**Health utility—change from baseline EQ-5D-5L**	6	0.14 (0.24)	0.15 (0.20)	0.01 (-0.12 to 0.10)	0.86
	12	0.18 (0.25)	0.07 (0.24)	0.11 (-0.01 to 0.24)	0.07
GROC*^ **(Mean rank)**	0	23.65	37.04	N/A	0.001
	6	31.63	32.39	N/A	0.83
	12	30.02	34.05	N/A	0.31
**Satisfaction (10cm VAS)**	12	9.64 (0.73)	9.55 (0.76)	0.09 (-0.30 to 0.47)	0.65
**Number of physiotherapy appointments**	12	5.03 (1.60)	4.52 (2.20)	0.51 (-0.45 to 1.84)	0.29
**Adverse events**	12	1 non-serious	1 non-serious	N/A	N/A
**Medication change** ^$^*(Mean rank)	12	32.77	32.21	N/A	0.87
**Post-injection ROM gain (degrees)**	0	13.40 (11.55)	14.38 (16.02)	0.97 (-5.97 to 7.91)	0.78
**Post-injection ROM gain at pain onset**	0	31.05 (29.85)	38.51 (30.87)	7.47 (-7.70 to 22.64)	0.33
**Post-injection worst pain reduction VAS**	0	29.91 (26.93)	38.51 (29.70)	8.61 (-5.54 to 22.76)	0.23
**Post-injection av. pain reduction VAS**	0	23.36 (24.67)	28.82 (23.85)	5.44 (-6.70 to 17.58)	0.37
**Usual days wait for appointment** ^@^	0	119.0 (28.59)	356.42 (113.08)	237 (261.96 to 212.79)	<0.001

* Mann-Whitney U test,

^ 5 point scale; 1 = completely recovered to 5 = much worse,

^$^ categories of pain medicine use at 12 weeks; 1 = stopped, 2 = reduced, 3 = same, 4 = increased,

^+^ p values form t-Tests, unless otherwise stated,

^@^ Usual waiting list duration (in days at the time of injection) for an appointment with physio and surgeon

### Health economic evaluation

**Fig 3:**
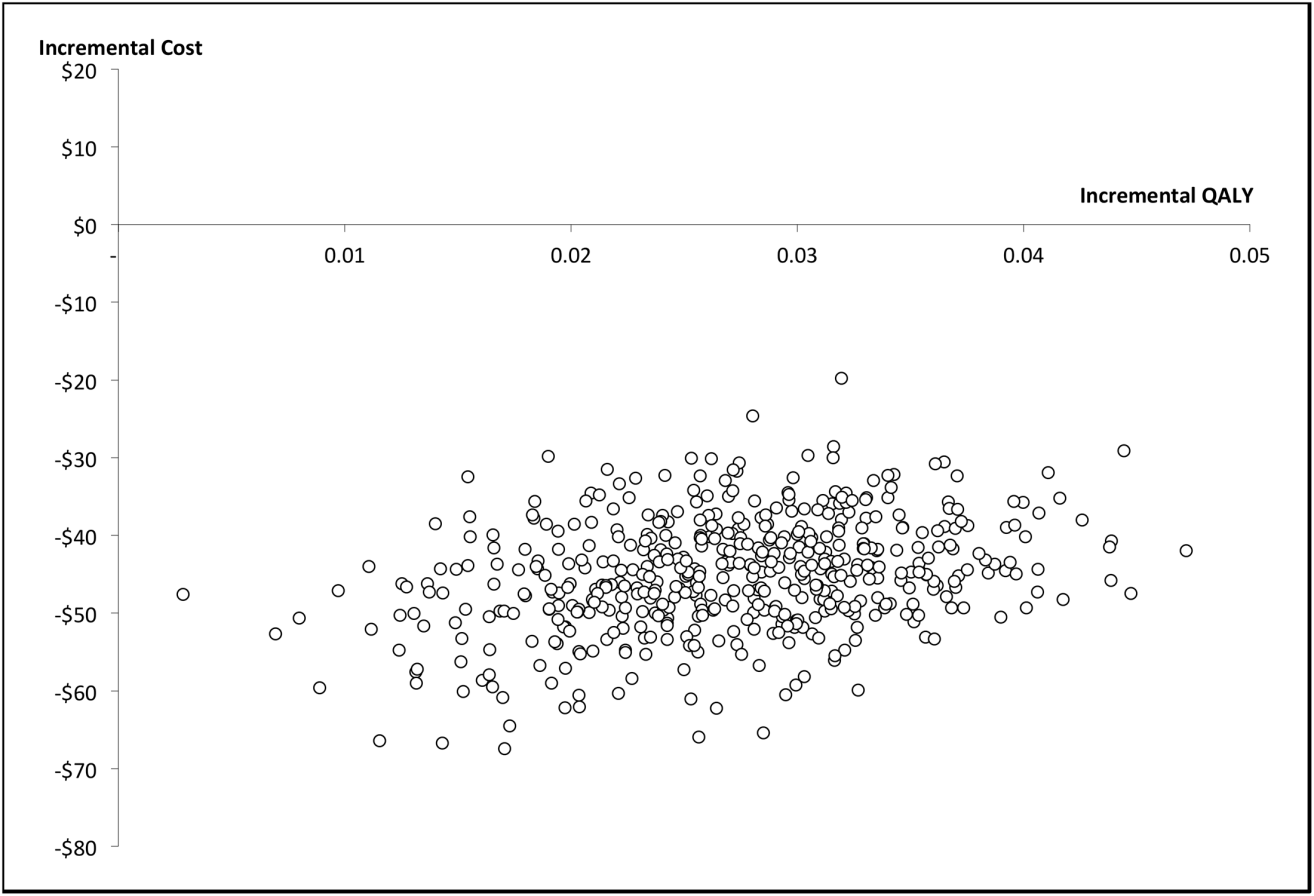
Plot of the first 1,000 bootstrapped estimates. Shows the incremental cost estimates per consultation and the incremental QALYs for the physiotherapist. All estimates place the physiotherapist as the dominant option with greater QALYs and cost saving.

Labour costs per 30-minute consultation period were calculated to be $86.99 for the surgeon, $32.04 for the injecting physiotherapist and $29.21 for physiotherapists providing post injection treatment. As there was no appreciable difference between the groups in change of medicines usage (Table 3), this cost variable was omitted from the calculation. Results of the health economic analysis are contained in Table 4. The base case calculation (from the health funder perspective over a 12 week time horizon) reveals that the physiotherapist is the dominant option; the physiotherapist is both less expensive, and as or more effective than the orthopaedic surgeon. One-way sensitivity analyses (Table 4) reveal the stability of the dominant result for physiotherapist injection with doubling of the physiotherapist consultation time, increased physiotherapist remuneration and with incorporation of additional staff for the orthopaedic clinic. The first 1,000 bootstrapped estimates plotted in Fig 3, all fall within the dominant south east quadrant of the plot demonstrating that the effect was consistently better and at lower cost compared with the orthopaedic surgeon. From the hospital perspective, the physiotherapist was slightly more expensive than the orthopaedic surgeon (incremental cost of $29) due to an Australian Government payment (Medicare) for services provided by doctors, which effectively subsidises the orthopaedic surgeon for the hospital. However, the physiotherapist also produced a slightly greater incremental QALYs score over the 12 week period (0.03), resulting in an ICER of $989. This indicates that for every additional QALY gained, the cost to the hospital is $989. This result is well below a conservative willingness to pay threshold (WTP) of AUD$50 000 per QALY [69], indicating that the physiotherapist is still cost effective.

**Table 4:** Health economic evaluation; base case and one-way sensitivity analyses.

Base case calculation	Physio	Surgeon	Increment	ICER*, Mean (95% CI)
Labour cost (30min)	$32.04	$86.99	N/A	
Base case effect—QALYs mean (SD)	0.16 (0.05)	0.13 (0.06)	0.03	Dominant
Base case total cost mean (SD)	$179 (46.62)	$214 (66.42)	-$35	
**One-way sensitivity analyses**				
Hospital perspective				
- include Medicare rebate^	$179	$150	$29	$989 ($365 to $1839)
Physiotherapist pay level				
- increase one grade	$182	$214	-$32	Dominant
Clinic support staff				
- orthopaedic clinic nurse	$179	$238	-$59	Dominant
Physiotherapist consultation time				
- Increase to 60 minutes	$211	$214	-$3	Dominant

*10,000 sample bootstrapping,

^ Federal Government Medicare rebate Item 104 ($64.20) only applicable to doctors, ICER: incremental cost effectiveness ratio. QALY: quality adjusted life year.

## Discussion

This is the first study to compare outcomes following the prescription and delivery of corticosteroid injection for the management of shoulder pain by a physiotherapist with the ‘gold standard’, the consultant orthopaedic surgeon. Results demonstrate similar [50] to more [49] improvement in total SPADI scores compared with previous studies of corticosteroid injection in a similar population, suggesting good effect size and that external validity was maintained with the inclusion criteria, which required participants to be selected for injection by two clinicians. There were no statistically significant or clinically important differences between the surgeon and the physiotherapist at six or 12 weeks, with mean differences below minimal clinically important difference (MCID) values for SPADI [47], pain VAS [52], and very small differences on other secondary outcomes (Table 2). The results also demonstrate that clinical outcomes measured by the SPADI were not inferior when the intervention was delivered by the physiotherapist compared with the orthopaedic surgeon at both the 6 and 12 week follow-up periods. Furthermore, the physiotherapist was less expensive than the surgeon. From a clinical and health services perspective, these findings indicate that for conditions such as shoulder pain that may not require surgery, care may be enhanced and expedited with physiotherapist prescription and delivery of injections if clinically indicated. This model creates potential for a seamless care pathway, as a physiotherapist also possesses skills in exercise prescription, which is also recommended within the management of these conditions [32, 37].

Shoulder pain poses a diagnostic and management conundrum for clinicians and researchers. Current evidence suggests that stating an exact pathoanatomical diagnosis may be misleading due to factors such as the inaccuracy of clinical assessment procedures [70] and a lack of correlation between radiological signs of rotator cuff deficiency and patient symptoms [71, 72]. In this trial we have attempted to take a balanced evidence-based perspective of shoulder pain, which includes the following considerations: that subacromial impingement comprises a variety of potential contributory factors [32] including such items as bone shape, rotator cuff pathology, acromio-clavicular joint changes, thickening or bursitis of the subacromial bursa and biomechanical factors such as scapula control [31, 73]; that subacromial impingement is likely to be the most common form of shoulder pain [31, 32]; that subacromial impingement is best diagnosed using clinical judgement and a variety of clinical tests combined with subjective history taking [30, 32, 74, 75]; that subacromial pain should generally be treated non-surgically [32], and that subacromial corticosteroid injection is an evidence-based recommended treatment option [32, 35, 36, 38, 39, 45]. Studies involving rat models have provided contradictory information about the impact of corticosteroid in the vicinity of the rotator cuff with one study reporting tendons to be weaker at 3 weeks post injection [76] and another reporting no lasting deficit [77]. Furthermore in humans one study simultaneously reported that 17% of subjects had a full thickness cuff tear 12 weeks after injection and that injection significantly improved symptoms [78]. Such aspects of shoulder histopathology, diagnosis and management highlight important and ongoing research challenges that this study does not attempt to answer. Rather, the aim of this study was to take a common and evidence-based treatment option for subacromial impingement [32] and investigate whether a physiotherapist might provide a more efficient service delivery model. Although conducted in the Australian health system, the findings have relevance to musculoskeletal service delivery internationally. Whilst already available in the UK, a physiotherapy-led model of care has not previously been subjected to robust investigation or comparison with a consultant orthopaedic surgeon. Our results validate these models of care, as it appears that corticosteroid injection provided by a trained physiotherapist is safe and effective.

This study has addressed some inadequacies of previous research comparing corticosteroid injection delivered by physiotherapists to that delivered by medical practitioners. Previous studies have not been specific regarding the inclusion of shoulder conditions or injection site [79–81], or injection was only a small element within a multi-modal treatment arm [81], or the profession of the individual delivering the injections was unclear [82], or sub-consultant doctors provided the control group [81, 82]. One factor that is common between past studies [79–81, 83] and our current study, however, is the absence of any reported serious adverse events. Our study is therefore the first to investigate and demonstrate safety, clinical efficacy, cost benefit and patient satisfaction, with physiotherapist prescribing and injection compared to a surgeon, for the management of shoulder pain. Furthermore, in previous musculoskeletal professional substitution research, professional equivalence has been claimed on the basis of a non-significant result. This is not justified because it may result from a lack of power or include confidence margins that extend beyond a clinically acceptable limit [63]. Non-inferiority demonstrates equivalence in the relevant one sided-confidence interval (in this case the upper limit as higher scores on SPADI indicate a worse outcome) supporting our claim that care from the physiotherapist is “not worse” or “at least as good” [63] as that from the orthopaedic surgeon. This approach is common in the pharmaceutical literature but to our knowledge this is the first study to apply non-inferiority to a professional substitution hypothesis.

We did not record the exact time each clinician spent with patients, however both clinicians worked to the same clinic schedule of 30-minute consultation times. We consider our assumption of equal overall costs of a physiotherapist clinic compared to an orthopaedic surgeon clinic to be conservative because in practice consultant clinics tend to have greater support staff such as nurses. Our measure of the usual waiting time to see the orthopaedic surgeon is also conservative because hospital data is retrospectively calculated from the waiting time of patients seen each month. As lower priority patients remain on the waiting list without being seen, true prospective waiting times may be much longer. The physiotherapist in this trial acted independently regarding decisions and delivery of corticosteroid injection, yet outside the UK this level of autonomy is generally beyond the scope of physiotherapy practice. Therefore, the findings of this study have policy implications for medicines legislation and health funding. The potential for public healthcare benefit from innovative new models, can only be realised if the legal and funding barriers to its implementation are removed. Our expectation is that optimum service efficiency for this patient population will result from a multidisciplinary team, which includes general practitioners, physiotherapists, orthopaedic surgeons and rheumatologists working autonomously within an integrated outpatient musculoskeletal pathway. Details such as the optimal proportion of physiotherapists compared with doctors in such a pathway would depend upon local demand variables, and further research is needed to provide a wider societal perspective of the health economic impact of professional substitution within such a pathway.

This study has some limitations. Firstly, having only one clinician in each treatment arm potentially reduces the generalisability of results. This situation was a direct consequence of the innovative nature of the research; prescribing and injecting by physiotherapists is beyond Australia’s legislative framework, and the physiotherapist (DM) had to demonstrate competency and experience by drawing upon overseas (UK) experience. In the absence of domestic legislative, curriculum or education frameworks for prescribing and injection by physiotherapists, additional suitably qualified physiotherapists were not available. Secondly, while adequate, our sample size was not large which allowed us to balance the resource and time challenges associated with conducting pragmatic clinical research in a public outpatient setting. Our use of a repeated measures design and the GEE approach helped provide an increased and sufficient power to detect non-inferiority (such as the achieved scenarios B and C in Fig 1). Confidence in our results is further strengthened by the absence of very wide confidence intervals (scenarios E or F Fig 1) associated with an inadequate sample size. Furthermore, greater numbers of participants would only decrease the confidence intervals and strengthen our non-inferiority conclusion (such as Scenarios A or D in Fig 1). Thirdly, we chose a 12-week endpoint as prior research suggests the effect of corticosteroid is greatest in the short-term [35] and whilst we feel this time period was adequate to test our hypothesis, a longer-term follow up could be considered in future research. A fourth relates to post-injection physiotherapy. Whilst steps were taken to remove bias from this phase of treatment and the number of physiotherapy sessions were reported, we did not test the blinding of the treating physiotherapists, or monitor the exact home exercises given or participants’ compliance with advice and exercises. More detailed monitoring of these factors, could provide more information about factors potentially impacting each participant’s outcome. Finally, our findings apply to a shoulder pain population and extrapolation to other orthopaedic or musculoskeletal populations should not be assumed. Previous research has suggested physiotherapy care fares well in comparison to usual orthopaedic care in mixed, knee, hip and back pain populations [28, 81, 82, 84], however these studies have design limitations and therefore validation of our findings in other patient groups may help clarify optimal service delivery models.

## Conclusions

A suitably qualified physiotherapist is able to prescribe and deliver corticosteroid injection for shoulder pain as least as effectively, and at less expense than a consultant orthopaedic surgeon. In the UK, where existing laws permit this practice, these findings support the employment of physiotherapists to provide these services. These findings also suggest that policy makers and service providers elsewhere should consider adopting this model of care.

## Supporting Information

S1 File Dataset. (XLS)

S2 File CONSORT checklist. (DOC)

S3 File Protocol. (DOC)
