# Supplementary material for: Increasing Capacity for the Treatment of Common Musculoskeletal Problems: A Non-Inferiority RCT and Economic Analysis of Corticosteroid Injection for Shoulder Pain Comparing a Physiotherapist and Orthopaedic Surgeon
Source: PLoS One. 2016 Sep 15;11(9):e0162679. doi: 10.1371/journal.pone.0162679 (PMC5025143; doi:10.1371/journal.pone.0162679)
Supplement: S3 File — (DOC) [file pone.0162679.s003.doc]

**RESEARCH PROTOCOL**

**HREC Reference HREC/12/QGC/30**

**SSA Reference SSA/12/QGC/97**

**Universal Trial Number (UTN) U1111-1130-6944**

**Title: An experienced physiotherapist prescribing and administering corticosteroid and local anaesthetic injections to the shoulder in an Australian orthopaedic service: a non-inferiority RCT.**

**Date:** 19 July 2012

**Version:** 3

**Principal Investigators**:

Darryn Marks

MSK Program Manager, OPSC Clinical Leader GCHSD

Professor Philip Conaghan

NIHR Leeds Musculoskeletal Biomedical Research Unit,

University of Leeds, UK

**Co-investigators**:

Dr Leanne Bisset

Principal Research Fellow GCHSD / Griffith University

Dr Michael Thomas

Orthopaedic Surgeon GCHSD

Dr Shaun O’Leary

Research Fellow OPSC / University of Queensland

Dr Tracy Comans

Research Fellow, Centre for Applied Health Economics School of Medicine Griffith University

Dr Shu Kay (Angus) Ng

Associate Professor (Biostatistics), School of Medicine,

Griffith Health Institute, Griffith University

**Contents**

|  |  |
| --- | --- |
| SYNOPSIS  OBJECTIVES  BACKGROUND  STUDY PROGRAMME  Design  Participants  Outcome Measures  Interventions  Procedures  Blinding  Randomization  Statistical analysis  GOVERNANCE AND SAFETY  PLACE OF STUDY  LENGTH OF STUDY  BENEFITS  DECLARATION  BUDGET  LIST OF FORMS  REFERENCES | 3  3  3  12  12  12  13  14  15  17  17  17  18  20  20  20  20  20  21  22 |

**SYNOPSIS**

Orthopaedic service delivery models involving trained physiotherapists injecting corticosteroid and local anaesthetic for musculoskeletal pain, are common in the UK. Laws and regulations do not normally permit physiotherapists in Australia to prescribe or inject medicines. This presents a large service gap in comparison to successful models of care in the UK. It also raises questions about the economic impact of the different service models.

Using an Australian physiotherapist who trained and practised prescribing and injecting in the UK, and in the clinical context of shoulder pain, this research aims to investigate the feasibility of a physiotherapist prescribing and delivering medicine by injection in an Australian setting.

This research will contribute to the evidence on advanced physiotherapy practise, economic impacts of shoulder pain and it’s service delivery models and also professional role boundaries. It will help inform service providers and policy makers locally and nationally.

# RESEARCH OBJECTIVES

This research aims to investigate the feasibility of a physiotherapist prescribing and delivering medicine by injection in an Australian setting.

**Primary objectives**

To discover whether:

1. there is agreement between the physiotherapist and the surgeon regarding selection of shoulder patients appropriate for subacromial injection.
2. care (subacromial corticosteroid and local anaesthetic injection) delivered by the physiotherapist is no worse than the same type of care delivered by the orthoapaedic surgeon, with respect to safety, access, effectiveness and efficiency.

**Secondary Objectives**

1. To measure the economic burden of shoulder pain, for patients referred to a public orthopaedic service.
2. To explore changes in shoulder pain and range of movement immediately following shoulder injection, as predictors of longer term response to injection.

**BACKGROUND TO THE PRIMARY OBJECTIVES**

***Service delivery models vary***

Healthcare delivery models involving trained allied health professionals selecting patients then delivering corticosteroid and local anaesthetic injections for musculoskeletal pain, are common in the UK. Peer reviewed and health policy publications have reported these models to be clinically and cost effective. Furthermore, the Medical Defence Union (UK) has reported that they are not aware of any problems related to prescribing or medicines supply by allied health professionals (Allied health professions prescribing and medicines supply mechanisms scoping project report, Dept Health, UK, July 2009).

Across Australia, including in Queensland, physiotherapists are not normally permitted to prescribe or administer medicines by injection. This presents a large service gap in comparison to successful models of care in the UK. Using an Australian physiotherapist who trained and practised prescribing and injecting in the UK, in the clinical context of shoulder pain, this research will investigate the feasibility of a physiotherapist prescribing and delivering medicine by injection in an Australian setting.

***Shoulder disorders are common***

Shoulder pain with concomitant limitation of movement is a common problem, with a prevalence of 20 - 33% in the general population (McBeth & Jones, 2007; van der Windt, Koes, deJong, & Bouter, 1995; Vermeulen et al. 2006) and as high as 46% in some sports (Kibler & Safran, 2005). The Gold Coast Health Service District (GCHSD) Orthopaedic Department received approximately 4800 new referrals in 2010/2011. After knees, shoulders were the largest referral category (16%). Long waiting lists are common within the Gold Coast Health Service District. As at 01.12.2011 the median waiting time for new orthopaedic referrals graded as non-urgent was 275 days for both Category 2 and Category 3. Service data also indicates that the majority of orthopaedic referrals are managed conservatively, with only approximately 35% of referrals resulting in a surgical episode (GCHSD 2011).

***There is a common group of shoulder disorders***

Subacromial Impingement Syndrome (SIS) has been described as the most common cause of shoulder pain, with multifactorial origins, including anatomical and mechanical factors, rotator cuff disease, glenohumeral instability or restriction, postural and muscle imbalance (Lewis et al 2001). Glenohumeral capsulitis appears to be a separate pathological process (Bunker et al 1995) to other causes of SIS.

There is some evidence supporting the use of clinical tests to diagnose SIS. Michener (2009) investigated 5 common clinical tests for SIS (Neer’s Impingement, Hawkins-Kennedy, Empty Can or Jobe, Painful arc, External rotation resistance test) against a reference standard of intra-operative findings and found that 3 or more positive tests out of the 5 helps confirm the diagnosis of SIS, while less than 3 positive helps rule out SIS.

***There is support for the use of corticosteroid injection for shoulder pain***

While many different shoulder management strategies exist, high level evidence does not clearly support any one treatment. Relevant Cochrane reviews provide some limited evidence to support the use of corticosteroid injection, physiotherapy and oral steroids in non-traumatic, non-instability based shoulder pain:

• Corticosteroid injection for shoulder pain (Buchbinder 2009): Subacromial corticosteroid injection for rotator cuff disease and intra-articular injection for adhesive capsulitis may be beneficial although their effect may be small and not well-maintained.

• Physiotherapy for shoulder pain (Green 2010): There is some evidence from methodologically weak trials to indicate that some physiotherapy interventions are effective for some specific shoulder disorders. The results overall provide little evidence to guide treatment.

• Oral steroids for adhesive capsulitis (Buchbinder 2009): oral steroids provides significant short-term benefits in pain, range of movement of the shoulder and function in adhesive capsulitis but the effect may not be maintained beyond six weeks.

• Acupuncture for shoulder pain (Green 2008): Due to a small number of clinical and methodologically diverse trials, little can be concluded from this review.

• Surgery for shoulder osteoarthritis (Singh 2010): No studies compared shoulder surgery to sham surgery, non-surgical modalities or placebo. Total shoulder arthroplasty seems to offer an advantage in terms of shoulder function, with no other clinical benefits over hemiarthroplasty.

• Surgery for rotator cuff disease (Coghlan 2009): No firm conclusions about the effectiveness or safety of surgery for rotator cuff disease. Three trials compared either open or arthroscopic subacromial decompression with active non operative treatment (exercise programme, physiotherapy regimen of exercise and education, or graded physiotherapy strengthening program). No differences in outcome between these treatment groups were reported in any of these trials.

Similar to many musculoskeletal conditions and perhaps in response to the lack of evidence supporting any one treatment strategy, many shoulder services pragmatically offer a variety of treatments. This multimodal approach is advocated by the UK National Institute of Clinical Excellence, whose national clinical guidelines for the management of Osteoarthritis in Adults (2008), recommends the use of combined physical and pharmaceutical measures, including intra-articular corticosteroid injection as an adjunct to other treatment for the relief of moderate to severe pain.

Crawshaw (2010) in a pragmatic randomised controlled trial recently tested this multimodal approach by comparing corticosteroid injection combined with exercise and manual therapy to exercise and manual therapy alone, in 232 patients with subacromial impingement syndrome. The combined injection and physiotherapy group was significantly better at on the primary outcome measure (SPADI) at week 1 and week 6, however there was no significant difference by week 12. There was still a higher complete recovery rate in the combined group at week 12. At week 24 more patients in the exercise only group continued to take painkillers and went on to seek an injection after their 12 week course of treatment ended.

***Corticosteroid injection may be a cost effective treatment***

The need for, and lack of, quality economic evaluation within existing shoulder literature, is summarised in a recent review of economic evaluations in shoulder pathologies (Kuye et al 2011). This work concludes that “future health care resource allocation will likely be based on the economic feasibility of treatments” and that “the current state of the literature is poor”. One of few health economic analyses of interventions for shoulder pain (James et al 2005), in a pragmatic RCT of 207 unilateral shoulder pain patients randomized to either physiotherapy or GP injection with 6 months follow up, demonstrated similar clinical outcomes across the two groups, however corticosteroid injections were more cost effective. The total mean costs, per patient, were £71.28 for the injection group and £114.60 for the physiotherapy group.

***Existing Queensland Health physiotherapist models have limitations***

The Gold Coast orthopaedic department directs many non-urgent shoulder referrals to the Orthopaedic Physiotherapy Screening Clinic and Multidisciplinary Service (OPSC). The OPSC began at GCHSD in 2007 and represents one arm of a state-wide service, each with unique site-specific characteristics. Orthopaedic referrals are viewed and categorised by orthopaedic consultants, who pass to the OPSC, those referrals that may benefit from further conservative management. Post graduate trained Clinical Leader Physiotherapists work at an advanced level in assessing these patients, requesting imaging (protocol initiated) and referring patients for ongoing management either with orthopaedics or with the OPSC multidisciplinary team. On the Gold Coast, approximately 65% of OPSC patients are successfully managed to discharge without the need for an orthopaedic appointment. As at 01.12. 2011 the median waiting time for new orthopaedic referrals graded as non-urgent was 275 days for both Category 2 and Category 3. In contrast the median waiting times for OPSC were 29 days for Category 2 and 54 days for Category 3 referrals (GCHSD 2011).

Gold Coast OPSC sees a high percentage of shoulder referrals, many of whom receive injection as part of their non-surgical management. Across Queensland OPSC sites, when OPSC clinical leaders identify patients likely to benefit from injection, several access routes are currently available:

1. Patients can be returned to the orthopaedic waiting list to be seen at a later date by the orthopaedic department.

2. OPSC Clinical Leaders can approach orthopaedic staff ad hoc, and ask whether they have time and are comfortable delivering an injection to an OPSC patient, or referring that patient for an injection.

3. OPSC clinical leaders can attempt to contact the patient’s GP to ask whether they would consider referring the patient for an injection.

These management approaches result in delays to optimal treatment (i.e. injection) with possible prolonged wait times as patients are returned to orthopaedic waiting lists. Under present regulations, no member of the allied health team is permitted to prescribe, administer or specifically advise patients on medicines. This highlights a potential gap in service for patients who might benefit from further medicines management as part of their care. Instead of receiving timely medicines management alongside OPSC intervention, patients’ pharmaceutical needs may not be addressed, or care may be delayed while concurrent input from a separate medical service is sought. This may result in suboptimal care and therefore raises a broad question of whether care could be improved, through the provision of medicines management within the OPSC. For shoulder patients in the OPSC, service delivery might be improved if a suitably trained OPSC clinician were able to select suitable patients and provide these injections.

***UK physiotherapist models may offer more benefits***

Experience from overseas suggests that suitably trained physiotherapists might safely and feasibly provide an injection service for shoulder patients in the OPSC. In the UK, corticosteroid and local anaesthetic injections for musculoskeletal conditions are commonly performed by suitably trained allied health professionals who can act autonomously in assessing and selecting patients appropriate for injection, in the delivery of the injection and follow-up care. ACT Health in collaboration with the University of South Australia (2008) conducted the most recent Systematic Review of the literature in relation to the spectrum of physiotherapy advanced practice in musculoskeletal and found evidence that:

• Advanced physiotherapists can reduce the burden on medical staff by taking referrals more appropriate for conservative care

• Waiting lists can be reduced with the introduction of advanced physiotherapy roles

• Advanced physiotherapists “…utilise high level musculoskeletal diagnostic skills to ensure soft tissue injuries are accurately identified and managed….are less likely to miss significant injury and make inappropriate referrals than senior house officers….can be as effective as junior orthopaedic surgeons in assessment and management of new orthopaedic referrals”

• Services achieve high levels of patient satisfaction

• Significant cost benefits can be achieved

These findings suggest potential service delivery and economic advantages with an alternative service delivery model.

***Physiotherapists can make sound orthopaedic decisions***

There is evidence to suggest that Advanced Physiotherapy practitioners are able to make a diagnosis comparable with that of an Orthopaedic consultant, provide a management plan consistent with that of an Orthopaedic consultant, and can predict the need for a surgical consultation in musculoskeletal conditions (Oakes et al. 2009; Rabey et al. 2009; McKay et al. 2009; Oldmeadow et al. 2007; Hattam et al. 2004). However, many of these studies used a retrospective chart audit which has limitations regarding the quality of data reported. Furthermore, no study has investigated the level of agreement between an Advanced Physiotherapy practitioner and an Orthopaedic consultant in determining the need for corticosteroid injection in patients with shoulder pain.

***Some UK physiotherapists prescribe and administer medicines***

For the past 10 years corticosteroid and local anaesthetic injections have been performed in the UK by appropriately trained allied health professionals, including physiotherapists who can act autonomously in assessing and selecting patients appropriate for injection, in the delivery of the injection and follow-up care. Since 2000 Patient Group Directions have provided a legal mechanism permitting appropriately trained allied health professionals to select and deliver the appropriate medicine (Dept of Health 2009). Since 2006 physiotherapists, podiatrists and radiographers have been able to undertake non-medical prescribing training and then practise as supplementary prescribers (Dept of Health UK 2009). In addition, the English Department of Health is presently undertaking the second stage of a public consultation on proposals for legislative amendment to permit full independent prescribing by suitably trained physiotherapists and podiatrists.

In 2009 there were estimated to be approximately 3000 injection-trained physiotherapists in the UK (DoH 2009). Training is normally at Post-graduate Diploma or Masters level and ensures the physiotherapist can independently assess and diagnose the need for injection and/or aspiration, understands the relevant pharmacology and can safely deliver the injection and follow-up care (CSP 2011). Evidence of the success and acceptance of services involving physiotherapist injecting comes from a variety of peer reviewed publications and UK health policy documents:

• The UK Musculoskeletal Framework (DoH 2006) advocates the use of integrated musculoskeletal services for a variety of orthopaedic, rheumatology and pain service referrals, utilising extended scope physiotherapists (ESPs). ESPs in these services make advanced level clinical management decisions and often deliver injection therapy.

• A scoping project report into the patient and service need for UK allied health professionals to have greater prescribing autonomy (DoH 2009), acknowledged well established physiotherapist use of medicines / injecting and reported that “no serious incidents or case law relating to AHP medicines use have been reported to this project. The Medical Defence Union reported to the project that they are aware of no particular problems relating to prescribing or medicines supply by allied health professionals”

• Daker-White et al (1999) conducted an RCT of 481 patients referred to orthopaedics, which compared management by advanced physiotherapist, versus usual management by orthopaedic doctors. Physiotherapists delivered intra-articular injection in 16 of 197 cases, the doctors in 17 of 195 cases. Physiotherapists delivered intramuscular injection in one case and the doctors in seven cases. The study does not detail whether intramuscular injections were targeted non-articular soft-tissue injections (such as a subacromial injection) or intramuscular injection intended for systemic action. It also does not detail which joints intra-articular injections were delivered to. Results revealed that on the basis of patient centred outcomes, the physiotherapists were as effective as post-fellowship doctors and clinical assistant orthopaedic surgeons in the initial assessment and management of new referrals, and generate lower initial direct hospital costs.

• Crawshaw (2010) in a pragmatic randomised controlled trial compared physiotherapists injecting subacromial corticosteroid and local anaesthetic combined with exercise and manual therapy, to exercise and manual therapy alone, in 232 patients with subacromial impingement syndrome. The combined injection and physiotherapy group was significantly better at on the primary outcome measure (SPADI) at week 1 and week 6, however there was no significant difference by week 12. There was still a higher complete recovery rate in the combined group at week 12. At week 24 more patients in the exercise only group continued to take painkillers and went on to seek an injection after their 12 week course of treatment ended.

***Investigation in the Australian context is needed***

Health Workforce Australia (2011) has recently highlighted the need to “accommodate overlapping professional boundaries, or introduce new roles and extended scopes of practice that have been working effectively in comparable countries for decades”.

It can be argued that within the existing literature lies demonstration of the ability of physiotherapists to make decisions (including selection of patients for injection) and deliver care (including autonomously performing injections) at a standard comparable with that of orthopaedic doctors. Furthermore there is suggestion that this comparable service could be offered at lower cost. Patient and service need, alongside service data has been sufficient to drive physiotherapist role expansion in the UK. However relatively few controlled trials have been conducted and others argue the need for “further robust research to evaluate the expansion of extended scope roles” (Kersten et al 2007).

To date, the existing evidence has had minimal impact upon Australian care delivery models. In contrast to the UK where advanced allied health practice has flourished, regulatory structures and delivery models in Australia have not evolved to include any advanced allied health practice involving medicines. Potential cultural, political and professional reasons for this diversity in international practise are complex. Further research could help clarify the feasibility of allied health providing medicines management in the Australian context.

***There is a gap in the existing research***

The existing pragmatic Australian standard for selecting and delivering orthopaedic outpatients corticosteroid and local anaesthetic injection is the Orthopaedic Consultant. To date, no previous Australian service based or research trials of physiotherapist prescribing and medicine administration have been published. With respect to injection for musculoskeletal conditions including shoulder pain, no studies have yet directly compared

1. physiotherapist and orthopaedic surgeon patient selection for injection,

2. the outcomes of injections delivered by physiotherapists to those delivered by a surgeon in a particular patient group.

**Primary objectives**

To discover whether:

1. there is agreement between the physiotherapist and the surgeon regarding selection of shoulder patients appropriate for subacromial injection.
2. care (subacromial corticosteroid and local anaesthetic injection) delivered by the physiotherapist is no worse than the same type of care delivered by the orthoapaedic surgeon, with respect to safety, access, effectiveness and efficiency.

**BACKGROUND TO THE SECONDARY OBJECTIVES**

**1. The economic burden of shoulder pain for patients referred to an orthopaedic service:**

**The burden of shoulder pain in the Australian population and in the group of patients referred to Orthopaedics, is not presently known.**

Little is known of the cost burden of shoulder pain in the Australian population. No recent Australian population specific studies have investigated it’s direct healthcare or indirect costs. Two recent European studies reported varying total costs associated with shoulder pain, both studies found that a small group of patients were responsible for the bulk of total costs and that indirect costs of sick leave were the largest contributor to total costs.

- Kuijpers et al (2006) prospectively used a self administered 6 month cost-diary on 587 patients with a first episode of shoulder pain who presented to General Practice in the Netherlands. Mean costs were low at E689. 50% of these costs were attributed to the indirect cost of sick leave from paid work. They also found that a small proportion (12%) of the cohort accounted for 75% of the total costs.
- Virta et al (2012) directly tracked health records and found higher mean costs in a recent prospective cost-of-illness study of 204 Swedish shoulder patients presenting to General Practice or Physiotherapy at 3 primary health centres over a 6 month period. The cost of sick leave accounted for a large percentage of the total costs (84%), and a small group of patients produced most of the costs (in this case 20% of patients were responsible for 91% of the total costs and 44% of the healthcare costs). Mean annual total cost was estimated at E4139 per patient.

Economic burden in the subgroup of shoulder patients referred to orthopaedics has not previously been reported. The above data for general primary care shoulder populations demonstrates great variability in reported cost. Furthermore, true costs for an orthopaedic referred subgroup (such as that of the present study), may be diluted within a general primary care cohort.

**Understanding the economic burden of shoulder pain in patients referred to orthopaedics, will help evaluation of service delivery models:**

Waiting lists for initial outpatient appointments highlight that access to orthopaedic surgeons is problematic on the Gold Coast and more broadly in the Australian public healthcare system. Quantifying the economic burden of shoulder pain, for patients referred to orthopaedics is necessary in order to quantify the true cost of time spent on the orthopaedic waiting list. It is also a necessary baseline for evaluation of alternative care delivery models.

**There are many considerations in calculating the burden of shoulder pain:**

Measuring the burden of an illness is multifactorial and requires consideration of 1. direct costs - those directly associated with healthcare provided, 2. indirect costs, which include loss of productivity at work, around the home and the impact on others and 3. intangible elements such as pain and quality of life (Phillips et al 2006, Lubek 2003). To calculate the monetary value of shoulder pain, direct and indirect costs must be considered. These can be measured prospectively or retrospectively by interrogation of health records or through patient reports.

Period of recall in patient reported measures:

Variation exists within the published healthcare economic literature, regarding the recall period used for patient reported measures. The Outcome Measures in Rheumatology Clinical Trials (OMERAT) is an informal international network and research collaborative engaged in ongoing work to clarify contextual factors, evaluate existing measures and design at-work productivity measures for use in arthritis (Tang 2011 b, OMERACT 2012). OMERACT (2012) report that in present productivity measures, the requested recall period can vary from 2 weeks, to 2 years. The literature suggests many factors impact patient recall bias and that accuracy is sensitive to the subject matter being recalled, for example major life events have accurate recall over years, while in contrast acute pain levels or food intake may be better suited to recall periods of hours or days (Stulla et al 2005). Research specific to healthcare cost recall of musculoskeletal conditions has tended to use a period of weeks to months, with some recent and relevant studies reporting acceptable accuracy over a 3 month recall period:

Merkesdal et al 2005, Compared patient reported to healthcare funder data for RA patients every 3 months over an 18 month period and reported that patients report their productivity losses from sick leave and disability adequately.

Pinto et al (2011) reported on the comparison of patient reported and database records for healthcare costs including GP care, hospital care and medication for patients with hip or knee osteoarthritis. Over a 3 month recall period, visits, the majority of medications and overall costs reported by patients were consistent with databases.

Valuing productivity loss in unpaid activities:

Disease related changes in the productivity of unpaid activities is a form of indirect cost which ais difficult to quantify. For example, productivity whist undertaking housework, parenting, education or volunteer work, or burden upon formal or informal carers such as family or neighbours, can be impacted by a shoulder condition but there is no definitive way to monetise these impacts. The human capital approach is prone to underestimating the contribution of these intangible costs, willingness-to-pay methods have been used but these are complex, time consuming (Lubek 2003) and beyond the scope of the present study. No specific questionnaires exist to capture this information.

Valuing productivity loss in paid employment:

1) Absenteeism:

Traditionally absenteeism was the main measure of the productivity loss. The burden of absenteeism can be calculated from the societal perspective by the Human Capital Approach, which considers the value of lost production, or the Friction Cost Method which only considers that these costs only occur for a period required to replace the absent worker (Virta et al 2012, Phillips 2006). Absenteeism can be measured from employee records, records of doctor prescribed sick leave (Virta et al 2012), or patient reporting. Patient reporting is an acceptable method as previous research has found good agreement between self-reported absences and employer records with Spearman correlation coefficients of 0.79 for women and 0.75 for men (Ferrie et al 2005) shown in one study and 0.81 and 0.71 for hours missed over one and four week recall periods in another study (Kessler 2003).

2) Presenteeism:

More recently presenteeism or at-work productivity loss has increasingly been considered important. It describes reduced productivity in people who remain at work with an illness. When total reduced productivity costs are considered to be the sum of medical, pharmaceutical, absenteeism and presenteeism, Chen (2009) estimated presenteeism to account for over half of total productivity loss in arthritis sufferers. Burton (2006) found arthritis suffers had particular problems with physical aspects of work, in comparison to other aspects such as concentration or time management.

OMERACT contributors have published recent reviews of productivity measures including Tang et al (2011 a), which identified 11 measures of productivity in arthritis conditions. The Rheumatoid Arthritis Worker Instability Scale has ample validation in osteoarthritis but it is not a true measure of productivity. It measures the gap between ability and job requirements and may have prognostic value for job placement. Dedicated productivity scales with best utility for the population of the present study were:

- Work Productivity and Activity Impairment Questionnaire (WPAI): this has evidence of use in and generalisability, to many musculoskeletal conditions, is compatible with economic costing and easy to administer.
- Work Limitations Questionnaire (WLQ): this is the most widely used measure in musculoskeletal, it has excellent construct validity and the benefit of being expressed as a percentage of productivity loss in dual clinical and economic purposes.

**We propose a combination of measures to capture a baseline value for the cost burden of shoulder pain in our study population:**

To capture data about the costs of medical and related care, we have designed a direct and indirect healthcare costs questionnaire which we plan to administer with a 12 week recall period. We are presently unable to predict how many participants will be in paid employment. The literature to date suggests that the WPAI and the WLQ are best for our study population. Expert opinion suggests using a combination of measures including the WLQ (Beaton 2012). Therefore we propose to include both the WPAI and WLQ, alongside our direct and indirect costs questionnaire.

**2. Investigation of shoulder pain and range of movement immediately following injection, as predictors of longer term response to injection:**

Re-testing of shoulder clinical signs following the delivery of local anaesthetic and corticosteroid is commonly taught and practised following subacromial and other musculoskeletal injections. Advocates’ generally consider immediate or within-consultation post-injection changes, to be suggestive of the contribution of subacromial structures to a patient’s pain, and potentially suggestive of long-term response to the injection. Anecdotally, while few clinicians make ongoing management decisions based purely on this immediate response, it is common practise and therefore thought to contribute to the clinical decision making process. To date no studies have directly investigated the prognostic value of within-consultation shoulder clinical tests as predictors of a longer-term response to subacromial injection.

**Secondary Objectives**

1. To measure the economic burden of shoulder pain, for patients referred to a public orthopaedic service.

2. To explore changes in shoulder pain and range of movement immediately following shoulder injection, as predictors of longer term response to injection.

# STUDY PROGRAMME

## Design

Stage 1: Demographic and disease burden measurements. Level of agreement between two examiners (physiotherapist and surgeon who are blinded to each others decisions) regarding patient selection for injection, and forms the entry criteria for entry to stage 2,

Stage 2: Randomised Controlled Trial in which participants will see either the physiotherapist or the surgeon for their injection

***Participants***

Volunteers aged 18 and over with a referral to orthopaedics for shoulder pain, will be screened for inclusion through a clinical assessment with both the physiotherapist and the orthopaedic surgeon, who will be blinded to each others decisions (stage 1). Participants will be blinded to the profession of their examiner. Only volunteers independently deemed appropriate for 'subacromial injection today' by both the physiotherapist and the surgeon, will be invited to enter stage 2. Participants will be blinded to the profession of the clinician who delivers the injection.

**Stage 1 inclusion criteria**

- - Volunteers aged 18 years and over with a new GP referral to orthopaedics for shoulder pain

**Stage 1 exclusion criteria**

- - Previous consultation with either the research physiotherapist or the research orthopaedic surgeon for this condition
  - No XRay of the shoulder in the past 12 months

**Stage 2 inclusion criteria**

- Agreement between examiners on “would you provide subacromial injection today?”
- Participants who are able fill in the questionnaires and follow post-injection instructions

**Stage 2 exclusion criteria**

- Previous surgery to the involved shoulder
- Currently taking anticoagulant medication
- Need for prophylactic antibiotics with the injection
- Pregnancy or breastfeeding

***Outcome measures***

The shoulder pain and disability index (SPADI) has been shown to be a reliable and valid outcome measure (Beaton 96, Williams 95, Roach 91, Heald 97, Paul 04).

Participant global assessment of change from baseline will be recorded using a 5 point scale – complete recovery, better, same, worse, much worse - as used in a previous pragmatic shoulder therapy trials (Hay 03, Crawshaw 10).

*** primary outcome measure stage 1**

**^ primary outcome measure stage 2**

| **Category** | **Measure** | **From** | **Timing** |
| --- | --- | --- | --- |
| **Clinical and Demographic Characteristics** | Age, sex, employment status, hand dominance, uni or bilateral | Participant | Baseline |
|  | Imaging to date and key findings | Referral | Baseline |
|  | Duration of symptoms | Participant | Baseline |
|  | Past treatments and costs – physiotherapy, injection, surgery, other | Participant | Baseline |
|  | Medications | Participant | Baseline |
| **Baseline outcome measures** | Impact and costs questionnaire | Participant | Baseline |
|  | Work Productivity and Activity Impairment Questionnaire (WPAI) | Participant | Baseline |
|  | Work Limitations Questionnaire (WLQ) | Participant | Baseline |
|  | Shoulder Pain and Disability Index (SPADI) | Participant | Baseline |
|  | 10 cm VAS patient perception of pain over the past 3 days | Participant | Baseline |
|  | EuroQual-5D-5L | Participant | Baseline |
| **Stage 1** | | | |
| **Clinical agreement** | Are there any safety contraindications to injection? | Clinicians | At time of assessment |
|  | Are there any safety precautions regarding injection? | Clinicians | At time of assessment |
|  | *** Would you provide subacromial injection today?** | Clinicians | At time of assessment |
|  | Are there any other reasons to exclude this participant from stage 2 | Clinicians | At time of assessment |
| **Stage 2** | | | |
| **Safety** | Incidence of adverse events | Adverse event reporting mechanism | Collated throughout |
| **Access** | Current new appointment booking dates with Orthopaedics and OPSC | Research Assistant | At time of intervention |
| **Effectiveness** | **^Shoulder Pain and Disability Index (SPADI)** | Participant | 6 weeks  12 weeks |
|  | 10 cm VAS patient perception of pain over past 3 days | Participant | 6 weeks  12 weeks |
|  | Global Rating of Change Scale | Participant | 6 weeks  12 weeks |
|  | EuroQual-5D-5L | Participant | 6 weeks  12 weeks |
|  | Physiotherapy attendances post injection | eMR / Groupwise | 6 weeks  12 weeks |
|  | 10 cm VAS patient satisfaction with care | Participant | 12 weeks |
| **Economic** | Staff costs for each clinician | Standard pay scales | 6 weeks  12 weeks |
|  | Impact and costs questionnaire | Participant | Baseline |
|  | Work Productivity and Activity Impairment Questionnaire (WPAI) | Participant | Baseline |
|  | Work Limitations Questionnaire (WLQ) | Participant | Baseline |

In addition, two exploratory outcome measures will be collected before and immediately following injection:

1. active shoulder range of movement measured with a goniometer
2. VAS of pain during that movement

Although presently not validated in this population, they reflect commonly collected indicators of response in a clinical setting. This will allow us to explore their potential as predictors of longer term response to injection.

## Interventions

Stage 1: Examination. In no specified order participants will be examined separately once by the physiotherapist and once by the orthopaedic surgeon. Participants will be blind to the profession of their examiner and will be asked to not discuss their first assessment with the second clinician.

Stage 2: Subacromial corticosteroid and local anaesthetic injection by either the physiotherapist or the surgeon. Following randomisation, participants will see either the physiotherapist or the surgeon for their injection consultation, which will include gaining consent for the procedure according to usual hospital protocols. Participants will be blind to the profession of the clinician who provides the injection. Participants will receive usual post injection care.

Both groups will receive the following standard post injection advice for the week following injection (Crawshaw 2010):

- avoid activities that provoke pain
- avoid repetitive overhead activities
- stop sporting activities involving the arm

In keeping with standard practice, both groups will be offered physiotherapy, beginning one week post injection. The treating physiotherapist will provide care at their discretion from a selection of common treatments including advice, exercise and manual therapy techniques. The number of treatment sessions is at the discretion of the physiotherapist.

**Clinical Procedures**

The Physiotherapist and the Orthopaedic surgeon providing injections will

- - Prescribe and deliver the injection using aseptic technique. The injection procedure involves preparing and administering the following drug and dose schedule for an average sized adult. Technique includes a small aspiration (draw back of the syringe), to check that any fluid does not appear suspect (purulent) and that the needle is not in a blood vessel. If found, suspect fluid will be aspirated and sent for microscopy, culture and analysis, and the injection will not be given.
  - Decide and implement ongoing management

**Injection Procedure**

PATIENT SELECTION

- thorough clinical assessment, including seeking and incorporating the necessary information from referrals, previous reports, other professionals, imaging, pathology and the patient.
- establish whether there is clinical indication for subacromial injection.
- establish whether it is safe to offer subacromial injection, incorporating standard precautions, and considering any contraindications and clinical judgement.

PATIENT CONSENT

- explain the management options, including alternatives to injection
- discuss and answer questions in relation to risks and benefits of the injection, based on the information sheet already provided, and any other questions
- obtain and record verbal and written consent

PREPARATION FOR PROCEDURE

- position patient (sitting on couch or chair)
- ensure all equipment is available the room:

drugs, needles/syringes/wipes, sharps disposal, gloves etc

INJECTION TECHNIQUE

- wash hands before drawing up drugs
- mark needle insertion point on the skin with an aseptic needle sheath
- wash hands and apply non-sterile gloves
- clean skin with alcohol wipe and allow it to dry
- without contaminating the cleaned area, insert needle, draw back (aspiration check) as safety check and providing appropriate to proceed, administer the injection.
  - If safety aspiration reveals fresh blood, reposition needle and repeat process until needle is clear of vessel before injecting
  - If safety aspiration reveals suspect fluid, change syringe and aspirate fluid to be sent for microscopy/culture/sensitivity. Abandon injection.
- withdraw needle and apply pressure to site with cotton wool, when bleeding stops apply band aid, or alternative as clinically indicated
- dispose needles, syringes ampoules and vials in the sharps bin
- de-glove and wash hands

POST INJECTION

- remain mindful of any signs of adverse reaction
- re-test shoulder signs as clinically indicated
- provide advice upon post injection shoulder care and management
- explain (and highlight post injection information sheet) how to recognise adverse reactions and what to do if any arise.

**Medicines and dose schedule**

- - Betamethasone and lidoocaine will be drawn into the same syringe, prior to administration
  - Methylprednisolone will be used as an alternative corticosteroid if required, eg: betamethasone not available.

**Medicines and dose schedule**

| **Drug** | **Site** | **Dose** | **Volume** |
| --- | --- | --- | --- |
| Betamethasone (Celestone Chronodose)  Ampoule: 5.7mg/ml  AND  Lidocaine Hydrochloride 1% | Subacromial  Subacromial | 5.7mg  5ml | 6ml |
| **OR in the event that betamethasone is not available** | | | |
| Methylprednisolone Acetate (Depo-medrol)  Vial: 40mg/ml  AND  Lidocaine Hydrochloride 1% | Subacromial    Subacromial | 40mg  5ml | 6ml |

***Recruitment and screening***

The Research Assistant will identify appropriate referrals from orthopaedics, phone potential participants and administer appointment bookings in communication with orthopaedics. Upon arrival the Research Assistant will complete stage 1 consent form and baseline data forms with participants. Following stage 1, the research assistant will analyse clinician’s response (yes/no) to the primary stage 1 outcome measure “would you offer subacromial injection today? ”, to determine whether:

1. Both clinicians would offer injection, in which case the Research Assistant consents the participant for stage 2.

2. Neither clinician would offer injection, in which case, the participant sees surgeon for ongoing care and is not invited to participate in stage 2.

3. Only one clinician would offer injection, in which case the participant sees the surgeon for ongoing care and is not invited to participate in stage 2.

After consenting for stage 2, the research assistant will take pre-injection measures of the exploratory outcome measure. The participant will then take the top envelope from the randomised pile and go to the appropriately numbered room. The clinician will check that the participant has come to the room indicated on their randomisation card, gain written consent for the procedure, deliver the injection and record on the Treatment Form. The research assistant will then take post-injection exploratory outcome measures.

The surgeons assessment form and the treatment form will be scanned into EMR and form the clinical notes for the consultation.

***Blinding***

Clinicians:

Surgeon and Physiotherapist:

Clinicians will be blind to the assessment findings of the other clinician.

Treating Physiotherapist (post injection):

The treating physiotherapist will not be advised who delivered the injection and will be asked to not retrieve this information from patient records.

Participants:

The profession of the clinicians will not be disclosed to participants.

For stage 2 participants, this blinding will be assessed post injection when participants will be asked to tick whether they think they saw the physiotherapist or the surgeon.

Research Assistant:

The research assistant will not know which clinician is in each room and will therefore be a blind assessor of these post injection exploratory outcomes of range of motion and pain.

***Randomization***

In Stage 2, a computer generated (excel software) random number sequence will be used to randomise the order of treating clinicians. The sequence will be stratified by source of the patients (i.e. OPSC waiting list versus general orthopaedic waiting list). The randomisation sequence will be concealed in sealed opaque envelopes and administered by the Research Assistant.

## Statistical Analysis

All statistical analyses will be performed on an intention to treat basis using the statistical package SPSS (V19.0, IBM, New York, USA), with alpha level set at 0.05.

The analysis of the GEE will be conducted using SAS Statistical Software version 9.2.

**Stage 1:**  Agreements between the decisions from the two clinician assessors will be investigated using the proportion of observed agreement, the probability of chance agreement, and the kappa statistics with the 95% confidence intervals.

**Stage 2:** For continuous outcomes, normality of data will be assessed and parametric tests applied if normality is upheld. Treatments from the two clinicians will be compared over time (pre- to post-intervention) on an intention-to-treat basis using the generalised estimating equation (GEE) approach (Liang and Zeger1986) with an exchangeable working correlation structure to account for within-subject correlation for repeated measurements. With the GEE, a normal distribution with an identity link will be used for continuous outcome measures. The respective pre-condition baseline scores will be included as a covariate. The effects of time (within-group differences), practitioner (between-group differences), and practitioner by time interaction (between-group differences over time) will be included in all models and assessed using the Wald χ2 test. Model fit and assumptions will be checked were appropriate within the GEE framework. The GEE approach works well with missing observations on outcome measures, assuming that they are missing completely at random (MCAR). The validity of this assumption will be examined. If the MCAR assumption was violated, adjustment using weighted GEE (Hogan et al., 2004) will be performed. Appropriate post hoc tests will be conducted if significant main or interaction effects are identified from the omnibus analyses.

**Sample size:** Assuming α = 0.05, β = 0.2, and a standard deviation of 21.7 points of the change in scores from baseline, we estimate that 54 participants (27 per group) will be required to test for a comparative efficacy between the two treatments with a noninferiority margin of 15 points in the SPADI scores. The above parameter values for the power calculation are based on previous study on comparing the responsiveness for SPADI and several indices in patients receiving corticosteroid injection therapy (Ekeberg et al., 2010), where the minimum clinically important difference (MCID) for the SPADI is 23.1 points at 6 weeks. The calculation is conservative in the sense that it ignores the added power inherent in the repeated measures design. However, it is appropriate when treatment comparisons at the two individual time points (6 weeks and 12 weeks) are performed to assess the interaction treatment effect at different time after the injection. Allowing for a conservative drop-out rate of 15%, we will recruit a total of 64 participants (32 per group) for stage 2. In order to obtain this number, we anticipate needing 128 recruits into stage 1 because we estimate that 50% of stage 1 participants will be eligible to enter stage 2.

# GOVERNANCE AND SAFETY

**Adverse Reaction Management**

The post injection education sheet will advise participants of the usual post-injection responses and how to manage any adverse events. Response to adverse events will involve:

For urgent events, with signs and symptoms of anaphylaxis a standard procedure code blue will be called if the participant is in the hospital. Anaphylaxis is rare and usually occurs within the first 30 minutes post injection. Participants will remain in the department for at least 30 minutes post injection. If outside the hospital, emergency department attendance is recommended and this is stated on the post injection information sheet.

For non-urgent events, participants will be advised to contact us and seek GP consultation if required. They will be offered consultation with Dr Thomas (Supervising Orthopaedic Surgeon), who will assess and manage the situation with recording on the Adverse Incident Assessment Form.

**Adverse Reaction Reporting**

Participants will be instructed through their post injection information sheets to report any such concerns to the research team. Adverse events attributable to the trial are one of the secondary outcome measures, they will be monitored by project investigators and be communicated directly to the Gold Coast Health Service District Human Research Ethics committee and the data/safety monitoring committee. An adverse event reportable to the data safety monitoring committee will be defined as one that requires hospitalization or antibiotic treatment of confirmed or suspected infection.

**Data Safety monitoring**

The biostatistician will monitor the progress of this project through interim statistical analyses, and a data/safety monitoring committee will review any adverse events associated with the project.

Stopping rules will be applied to the following situations:

• Treatments found to be convincingly different

• Treatments found to be convincingly not different

• Adverse events too prevalent

**Competence and experience of the injecting physiotherapist**

The OPSC Clinical Leader who would prescribe and deliver corticosteroid and/or local anaesthetic injections in the proposed study, is an Australian trained physiotherapist who undertook further training and was employed as an Extended Scope Practitioner and then Consultant Physiotherapist in the United Kingdom NHS until August 2010. This individual has over 5 years of experience injection therapy practise. The following is a summary of this individual’s relevant training:

- Bachelor of Physiotherapy (Hons), The University of Melbourne 1994
- Master of Musculoskeletal Physiotherapy, LaTrobe University 2000
- Postgraduate Diploma in Injection Therapy, UK 2005
- Masters level module in X Ray interpretation, Sheffield Hallam University 2005
- External and in-house rheumatology training in blood test requesting and interpretation (2005 - 10)
- Non-Medical Prescribing Qualification, Sheffield Hallam University 2007

**Additional regulatory and governance arrangements**

Prescribing and medicines regulations

Through the office of the Chief Health Officer Approval under the Health Drugs and Poisons Act 1966, and subsequent Amendment to that Approval has been granted.

Gold Coast governance authorisation

GCHSD Credentialing according to the newly established statewide guidance for credentialing allied health professionals in advanced scope of practice activities.

The Physiotherapist has agreed with GCHSD Resuscitation Coordinator that no change to existing mandatory training is required.

The Physiotherapist has agreed with GCHSD Infection Control department that no additional training is required and the Injection Procedure has been agreed.

A period of supervised practice has been agreed with the Orthopaedic Department, consisting of a minimum of 7 injections. Sign off by the supervising Orthopaedic Consultant will be required prior to beginning data collection.

Accreditation of prescribing competence

Independent accreditation of prior prescribing and injecting competencies has been assessed and verified by the University of Queensland Centre for Safe and Effective Prescribing.

# PLACE OF STUDY

Gold Coast Health Service District

# LENGTH OF STUDY

3 Years

Start Date: 01 March 2012

End Date: 28 Feb 2015

|  | JAN | FEB | MAR | APR | MAY | JUN | JUL | AUG | SEP | OCT | NOV | DEC |
| --- | --- | --- | --- | --- | --- | --- | --- | --- | --- | --- | --- | --- |
| 2012 |  |  |  |  |  |  |  |  |  |  |  |  |
| 2013 |  |  |  |  |  |  |  |  |  |  |  |  |
| 2014 |  |  |  |  |  |  |  |  |  |  |  |  |
| 2015 |  |  |  |  |  |  |  |  |  |  |  |  |

White: Recruit research assistant, establish databases, physiotherapist accreditation

Light Grey: Participant recruitment and follow- up

Dark Grey: Data analysis and write up

# BENEFITS

The findings from this research will add to the evidence base which can help policy makers, regulators, professionals and the public decide whether it is feasible (safe, effective and efficient) for an appropriately trained physiotherapist to prescribe and administer medicines in certain situations. This alternative model of care delivery has the potential to reduce waiting times and improve access to appropriate care for patients who presently face long orthopaedic waiting lists. It offers the opportunity for improving patient flow, which will ultimately benefit both surgical and non-surgical orthopaedic pathways.

**DECLARATION**

The Principle Investigator, who is also the Physiotherapist delivering the injections in this proposed study, is presently seconded to a Program Manager post, funded through Queensland Health, Allied Health Workforce Advice and Coordination Unit (AHWACU) “Models Of Care” project funding. This research forms part of the work within that Program. AHWACU has no direct influence over the conduct of the research or its findings.

# BUDGET

| **Item** | **Source** | **Cost ($)** |
| --- | --- | --- |
| **Personnel**   - Principal Investigator 0.4 FTE HP5.2 for 2 years - Research Assistant 0.6FTE HP3.7 1yr - Co-investigators - Expert Consultation | Project Funding  Project Funding  Griffith University  In kind | 98926  56836 |
| **Equipment**   - Injectable drugs and syringes, including disposal - Photocopying: patient information and consent forms; screening assessment forms; standardised data recording forms = $100.00 - Postage of participant information forms, 6 and 12 week data collection forms (approx 163 X 60cents), including reply paid for 6 and 12 week outcomes (approx 64 X 60 cents) | Orthopaedic Dept  In kind  Physiotherapy Dept  In kind  Project Funding | 150 |
| **GRAND TOTAL** |  | **$156 412** |

# LIST OF FORMS

# Participant information and consent

# Corticosteroid and local anaesthetic injection information sheet

# Post injection information sheet

# Clinician assessment form

# Clinician treatment form

# Adverse reaction assessment form

# REFERENCES

ACT Health (2008). Physiotherapy Extended Scope Practice: Phase 1 Final Report.

Beaton DE, Richards RR. (1996) Measuring function of the shoulder. A cross-sectional comparison of five questionnaires. The Journal of Bone and Joint Surgery;78-A: 882-890.

Beaton D (2012). Email correspondence: Re query about worker productivity measures. 23.06.2012 12.52am.

Bellamy et al (2009). Cochrane Review: Corticosteroid injections for osteoarthritis of the knee.

Buchbinder R, Green S, Youd JM (2009). Corticosteroid injections for shoulder pain. Cochrane Database of Systematic Reviews 2003, Issue 1. Art. No.: CD004016. DOI: 10.1002/14651858.CD004016.

Buchbinder R, Green S, Youd JM, Johnston RV (2009). Oral steroids for adhesive capsulitis. Cochrane Database of Systematic Reviews 2006, Issue 4. Art. No.: CD006189. DOI: 10.1002/14651858.CD006189.

Bunker TD, Anthony PP. The pathology of frozen shoulder: A dupuytren-like disease. J Bone Joint Surg (Br) 1995; 77-B: 677-83.

Burton W, Chen C, Schultz B, Conti D, Pransky G, and Edington D. (2006). Disease Management. 9(3): 131-143. doi:10.1089/dis.2006.9.131.

Chen C, Edington D and Schultz A (2009). The cost and impact of health conditions on presenteeism to employers: a review of the literature. PharmacoEconomics. 27.5 (May 2009): p365.

Coghlan JA, Buchbinder R, Green S, Johnston RV, Bell SN (2009). Surgery for rotator cuff disease. Cochrane Database of Systematic Reviews 2008, Issue 1. Art. No.: CD005619. DOI: 10.1002/14651858.CD005619.pub2.

Croft P, Pope D, Silman A. The clinical course of shoulder pain: prospective cohort study in primary care. BMJ 1996;313:601-602.

Crawshaw D, Helliwell P, Hensor E, Hay E, Aldous S, Conaghan P

(2010). Exercise therapy after corticosteroid injection for moderate

to severe shoulder pain: large pragmatic randomised trial. BMJ 2010;340:c3037

Dakar-White, G. (1999). A randomised controlled trial. Shifting boundaries

of doctors and physiotherapists in orthopaedic outpatient departments. J Epidemiol Community Health 1999;53:643–650

Department of Health (2011). Consultation on proposals to introduce independent prescribing by Physiotherapists.

Department of Health (2009). Allied health professions prescribing and medicines supply mechanisms scoping project.

Department of Health (2006). A joint Responsibility. The Musculoskeletal Framework.

GCHSD Gold Coast Health Service District (2011). Internal orthopaedic and OPSC service reports.

Green S, Buchbinder R, Hetrick SE (2008). Acupuncture for shoulder pain. Cochrane Database of Systematic Reviews 2005, Issue 2. Art. No.: CD005319. DOI: 10.1002/14651858.CD005319.

Green S, Buchbinder R, Hetrick SE (2010). Physiotherapy interventions for shoulder pain. Cochrane Database of Systematic Reviews 2003, Issue 2. Art. No.: CD004258. DOI: 10.1002/14651858.CD004258.

Ferrie J.E et al (2005). A comparison of self reported sickness absence with absenses recorded in employers’ registers: evidence from the Whitehall II study. Occup Environ Med. 62: p. 74-79

Hattam, P. (2004). The effectiveness of orthopaedic triage by extended scope

physiotherapists. Clinical Governance Volume 9 • Number 4 • 2004 • pp. 244-252

Hay EM, Thomas E, Paterson SM, Dziedzic K, Croft PR. A pragmatic randomised controlled trial of local corticosteroid injection and physiotherapy for the treatment of new episodes of unilateral shoulder pain on primary care. Ann Rheum Dis 2003;62:394-399.

Heald SL, Riddle DL, Lamb RL. The shoulder pain and disability index: The construct validity and responsiveness of a region-specific disability measure. Physical Therapy 1997; 77:1079-1089.

Health Workforce Australia (2011). National Health Workforce Innovation and Reform Strategic Framework for Action 2011–2015

Hogan, J et al (2004). Tutorial in biostatistics: Handling drop-out in longitudinal studies. Stat Med 23:1455-1497.

James, M et al (2005). A cost consequences analysis of local corticosteroid injection and physiotherapy for the treatment of new episodes of unilateral shoulder pain in primary care. Rheumatology;44:1447–1451

Kelly, A.M., The minimum clinically significant difference in visual analogue scale pain score does not differ with severity of pain. Emerg Med J, 2001. 18:205-207.

Kersten, P, McPherson, K, George, S, Lattimer, V, George, S, Breton, A & Ellis, B

(2007). 'Physiotherapy extended scope of practice - who is doing what and why?'

Physiotherapy, vol. 93, pp. 235-242.

Kessler R.C., et al. (2003). The World Health Organization Health and Work Performance Questionnaire (HPQ). J Occup Environ Med. 45(2): p. 156-174

Kibler, B., & Safran, M. (2005). Musculoskeletal injuries in the young elite tennis player Climnica in Sports Medicine 19(4), 120-137.

Kuijpers, T., M. W. van Tulder, et al. (2006). "Costs of shoulder pain in primary care consulters: a prospective cohort study in The Netherlands." BMC Musculoskelet Disord 7: 83.

Kuye, I et al (2011). Economic evaluations in shoulder pathologies: a systematic review of the literature. J Shoulder Elbow Surg. 2011 Aug 22. [Epub ahead of print]

Lewis J, S, Green A, S, Deke S (2001). The Aetiology of Subacromial Impingement Syndrome. Physiotherapy. Volume 87, Issue 9, September 2001, Pages 458-469

Liang, K., & Zeger, S. (1986). Longitudinal data analysis using generalised linear models. Biometrika 73:13-22.

Lubeck DP (2003). The costs of musculoskeletal disease: health

needs assessment and health economics. Best Practice & Research Clinical Rheumatology

Vol. 17, No. 3, pp. 529–539.

MacKay, C et al (2009). Expanding roles in orthopaedic care: a comparison of

physiotherapist and orthopaedic surgeon recommendations for triage. Journal of Evaluation in Clinical Practice 15 (2009) 178–183

McBeth, J., & Jones, K. (2007). Epidemiology of chronic musculoskeletal pain Best Practice and Research in Clinical Rheumatology 21, 403-425.

Merkesdal, S., Ruof, J., Huelsemann, J. L., Mittendorf, T., Handelmann, S., Mau, W. and Zeidler, H. (2005), Indirect cost assessment in patients with rheumatoid arthritis (RA): Comparison of data from the health economic patient questionnaire HEQ-RA and insurance claims data. Arthritis & Rheumatism, 53: 234–240.

Michener, L et al (2009): Reliability and Diagnostic Accuracy of 5 Physical Examination Tests and Combination of Tests for Subacromial Impingement. Arch Phys Med Rehabil Vol 90, November 2009

National Institute of Clinical Excellence NICE (2008). Osteoarthritis. National clinical guideline for care and management in adults.

Oakes, H (2009). Orthopaedic shoulder clinic diagnosis and treatment plan audit. Clinical Governance.Vol. 14 No. 2, pp. 126-133

Oldmeadow, L. B., H. S. Bedi, et al. (2007). "Experienced physiotherapists as gatekeepers to hospital orthopaedic outpatient care." Medical Journal of Australia 186(12): 625-628.

OMERACT (2012). OMERACT 11 Pre-conference Paper for meeting May 2012.

Paul A, Lewis M, Shadforth MF, Croft PR, van der Windt DAWM, Hay EM. A comparison of four shoulder-specific questionnaires in primary care. Ann Rheum Dis 2004; 63:1293-1299.

Phillips, C. J. (2006). Economic burden of chronic pain. Expert Review of Pharmacoeconomics & Outcomes Research, 6(5), 591-601. doi:10.1586/14737167.6.5.591

Pinto D, Robertson MC, Hansen P, et al. (2011). Good agreement between questionnaire and administrative databases for health care use and costs in patients with osteoarthritis. BMC Med Res Methodol;11:45

Queensland Health, Allied Health Credentialing and Accreditation Clinical Governance Framework (2011). http://qheps.health.qld.gov.au/ahwac/content/clin_govhome.htm

Roach KE, Budiman-Mak E, Songsiridej N, Lertratanakul Y . Development of a shoulder pain and disability Index. Arthritis Care and Research 1991; 4:143-149.

The Chartered Society of Physiotherapy (2011). PD071: CSP expectations of

educational programmes in Injection Therapy for physiotherapists. Supporting Good Governance in Neurological and Musculoskeletal Injection Therapy

Rabey, M et al (2009). Orthopaedic physiotherapy practitioners surgical and radiological referral rates. Clinical Governance: Vol. 14 No. 1, 2009 pp. 15-19

Singh JA, Sperling J, Buchbinder R, McMaken K (2010). Surgery for shoulder osteoarthritis. Cochrane Database of Systematic Reviews 2010, Issue 10. Art. No.: CD008089. DOI: 10.1002/14651858.CD008089.pub2.

Stulla D, Kline Leidyb N, Parasuramanc B and Chassany O. (2005). Optimal recall periods for patient-reported outcomes: challenges and potential solutions. CURRENT MEDICAL RESEARCH AND OPINION. VOL. 25, NO. 4, 2009, 929–942.

Tang K, Beaton DE, Boonen A, Gignac MAM, Bombardier C (2001 a). Measures of Work Disability and Productivity. Rheumatoid Arthritis Specific Work Productivity Survey (WPS-RA), Workplace ActivityLimitations Scale (WALS), Work Instability Scale for Rheumatoid Arthritis (RA-WIS),Work Limitations Questionnaire (WLQ), and Work Productivity and Activity Impairment Questionnaire (WPAI). Arthritis Care & Research. Vol. 63, No. S11, November 2011, pp S337–S349

Tang K, Escorpizo R, Beaton DE, Bombardier C, Lacaille D, Zhang W, et al. (2011 b). Measuring the impact of arthritis on worker productivity: perspectives, methodologic issues, and contextual factors. J Rheumatol Aug;38(8):1776-90.

van der Windt, D., Koes, B., deJong, B., & Bouter, L. (1995). Shoulder disorders in general practice: incidence, patient characterisitcs, and management Annals of Rheumatic Diseases 54, 959-964

Vermeulen, H. M., Roing, P., Pobermann, W., le Cessie, S., Viet, V., & Thea, P. (2006). Comparison of high-grade and low-grade mobilization techniques in the management of adhesive capsulitis Physical Therapy 86, 355-368.

Virta, L., P. Joranger, et al. (2012). "Costs of shoulder pain and resource use in primary health care: a cost-of-illness study in Sweden." BMC Musculoskelet Disord **13**(1): 17.

Williams JW Jr, Holleman DR Jr, Simel DL. Measuring shoulder function with the shoulder pain and disability index. J Rheumatol 1995; 22: 727-732.
